# Real-Time Identification of Irrigation Water Pollution Sources and Pathways with a Wireless Sensor Network and Blockchain Framework

**DOI:** 10.3390/s20133634

**Published:** 2020-06-28

**Authors:** Yu-Pin Lin, Hussnain Mukhtar, Kuan-Ting Huang, Joy R. Petway, Chiao-Ming Lin, Cheng-Fu Chou, Shih-Wei Liao

**Affiliations:** 1 Department of Bioenvironmental Systems Engineering, National Taiwan University, Taipei 10617, Taiwan; mukhtar@ntu.edu.tw (H.M.); r06622004@ntu.edu.tw (K.-T.H.); d05622007@ntu.edu.tw (J.R.P.); chiaominglin@ntu.edu.tw (C.-M.L.); 2 Department of Computer Sciences and Engineering, National Taiwan University, Taipei 10617, Taiwan; ccf@csie.ntu.edu.tw (C.-F.C.); liao@csie.ntu.edu.tw (S.-W.L.)

**Keywords:** Internet of Things, pollution source trace, blockchain, GIS, pollution pathway, wireless sensor network

## Abstract

Real-time identification of irrigation water pollution sources and pathways (PSP) is crucial to ensure both environmental and food safety. This study uses an integrated framework based on the Internet of Things (IoT) and the blockchain technology that incorporates a directed acyclic graph (DAG)-configured wireless sensor network (WSN), and GIS tools for real-time water pollution source tracing. Water quality sensors were installed at monitoring stations in irrigation channel systems within the study area. Irrigation water quality data were delivered to databases via the WSN and IoT technologies. Blockchain and GIS tools were used to trace pollution at mapped irrigation units and to spatially identify upstream polluted units at irrigation intakes. A Water Quality Analysis Simulation Program (WASP) model was then used to simulate water quality by using backward propagation and identify potential pollution sources. We applied a “backward pollution source tracing” (BPST) process to successfully and rapidly identify electrical conductivity (EC) and copper (Cu^2+^) polluted sources and pathways in upstream irrigation water. With the BPST process, the WASP model effectively simulated EC and Cu^2+^ concentration data to identify likely EC and Cu^2+^ pollution sources. The study framework is the first application of blockchain technology for effective real-time water quality monitoring and rapid multiple PSPs identification. The pollution event data associated with the PSP are immutable.

## 1. Introduction

Illegal wastewater discharge due to rapid industrialization has resulted in heavy metal contamination in farmlands via irrigation channels. It is a severe threat to the environment, agricultural production, and public health [1,2,3]. Although rapid identification of irrigation water pollution sources and pathways (PSP) is key to managing irrigation water quality for agricultural production, it is an extremely difficult task in agricultural areas that are located within industrialized areas [4,5,6]. A real-time water quality monitoring network can collect water quality information at set (or at network) locations in real-time (or at regular intervals) and can provide monitoring data for both current status analysis and water quality trend forecasts. Potential pollution sources can then be identified [7,8], enabling the emergency disposal of pollutants in contaminated areas [9]. Moreover, a system that allows PSP tracing is essential to providing authorities with real-time documentation that narrows the scope of likely pollution sources for rapid identification and isolation of irrigation water pollution and pathways; and for protecting agricultural production environments. Furthermore, real-time identification and isolation of PSP are necessary for water quality management and control actions [6,8]. Pollution event data should be secured for further actions such as tracing and recording PSP without risk of data tampering.

Recently, blockchain technology has been widely utilized in many fields such as healthcare for medical data [10,11]; food production tracing [12,13]; and has been proposed for application to irrigation water resource management [6,14]. Blockchain technology provides significant security advantages such as user authentication through public keys and digital fingerprints, data immutability, and transaction transparency with traceability characteristics [15,16]. Since all transactions use hash functions and use hash values as fingerprints, a blockchain ensures that data cannot be changed. Critical information storage (e.g., pollution event data) and transactions can be delegated to the blockchain, while endpoint devices or platforms can remain ‘dumb’, untrusted, and require very little maintenance [17]. Blockchain technology’s robust consensus-based security architecture that does not require a central certifying authority also renders it particularly suitable for the authentication of ownership rights [17,18]. Accordingly, blockchain transaction records can reference uploaded and secured data [19,20]. Currently, the two most used blockchain transaction models are the unspent transaction output (UTXO) model and the account-based model. Just as blockchain technology used with Internet of Things (IoT) technology provides a way to trace a product’s lifecycle from raw materials used, to end production [21]; these technologies can be used to trace a pollution event from the pollution source, through its pathways, to the polluted area. Blockchain technology used with IoT and sensor networks, however, can potentially identify PSP. Moreover, pollution event data can be secured by blockchain technology.

Integrating effective real-time tools with real-time water quality monitoring data can improve the identification of irrigation PSP. Wireless sensor networks (WSN) have been developed using various sensors in different fields in order to provide high-quality real-time remote monitoring data [22] since traditionally conducted field investigations are often time-consuming and labor-intensive. In agriculture studies, WSN has been used for monitoring environmental conditions; scheduling irrigation based on real-time network data; controlling environmental conditions and parameters to improve cropping processes, and improving production quantity and quality [23,24]. A water quality simulation program using WSN data, however, can also be a useful tool to effectively predict point source pollution in irrigation channels since the location, time, and source (i.e., source tracing) of water quality contamination can be determined using sensor data [6,25,26]. Furthermore, a water quality sensor network with WSN can collect application-oriented data, and provide real-time information for agricultural and environmental monitoring [6]. Wireless sensor networks with blockchain technology then provide an effective way to identify and isolate PSP.

Still, a real-time irrigation water quality monitoring system with WSN, which is also a geographic information system (GIS), could simultaneously monitor irrigation water quality and quantity, spatially identify pollution sources, and track source locations. Recently, GIS, Global Positioning System (GPS) technology, and WSN have been utilized in various fields such as agriculture systems monitoring, flood risk analysis prediction, and urban systems monitoring [27,28]. This is because GIS technology is a powerful tool for spatial and temporal data analysis that has already been widely applied to identify contaminants in surface water [29,30,31]. Once a likely pollution area is identified with GIS, authorities can focus their investigation on the sources within the vicinity of the polluted wastewater discharge (e.g., specific factories).

Additionally, different deterministic, stochastic, and statistical models have also been used, at regionally and local scales, to identify various PSP through agricultural irrigation channels [4]. For example, the Water Quality Analysis Simulation Program (WASP), developed by the US Environmental Protection Agency, is a dynamic compartment-modeling program for aquatic systems. In the last two decades, WASP has been applied in several environmental studies to estimate impacts of nutrient loads on agricultural activities; or to examine the fate and transport of environmental pollutants [25,32]. The model, however, has been widely used in water quality simulation including surface water heavy metal concentration simulations [33,34,35]; and can be used to structure one-, two-, and three-dimensional models to help users interpret and predict water quality responses to natural phenomena and man-made pollution, for various pollution management decisions [36]. Users can also use the model to develop new kinetic and reactive structures. 

Industries have been occasionally implicated in discharging wastewater into irrigation channels in some of Taiwan’s agricultural areas [1]. Expressly, Lin et al. indicated that pollutants, such as heavy metals, may be delivered through irrigation channels into certain paddy fields in Taiwan. The aim of this study then is to develop a GIS integrated blockchain-based traceability system in order to identify PSP with a “blockchained” irrigation WSN. Furthermore, we simulated pollution concentration using WASP to trace wastewater discharge concentrations from highly likely pollution sources at irrigation units within our study area. The computational complexity analysis was done by using the big O notation.

## 2. Materials and Methods

**Figure 1:**
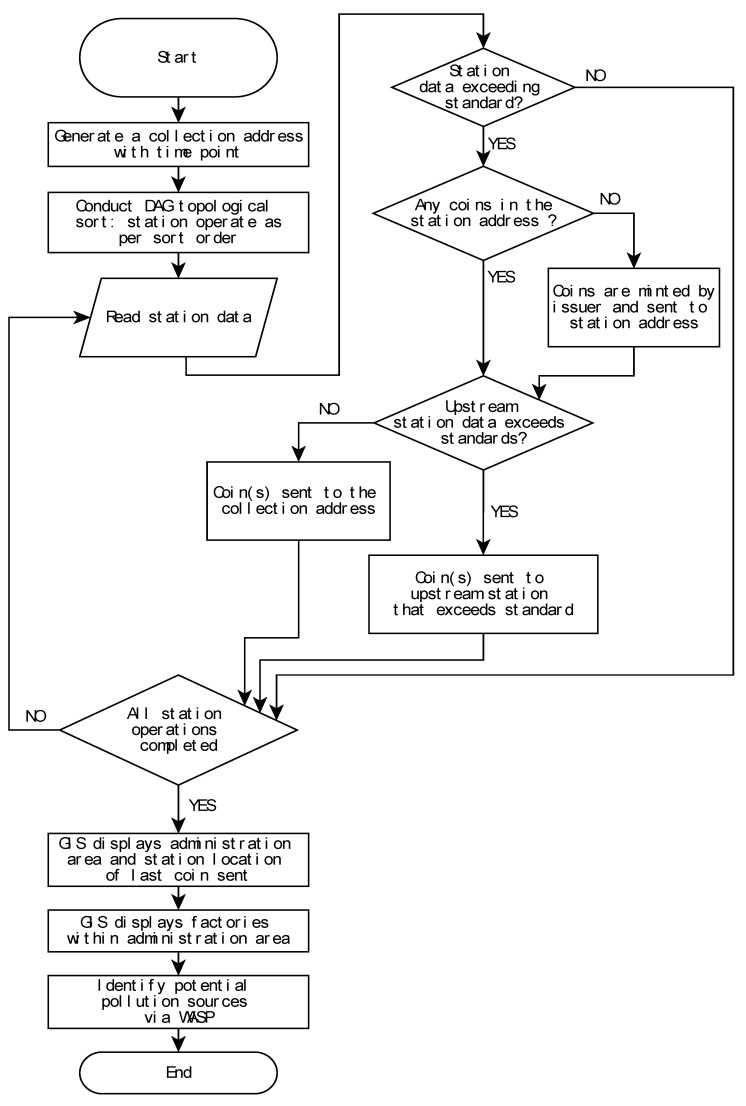
Flowchart of the data uploading process for the identification of pollution events and potential sources. Note: Directed Acyclic Graph (DAG); Geographic Information System (GIS); Water Quality Analysis Simulation System (WASP).

**Figure 2:**
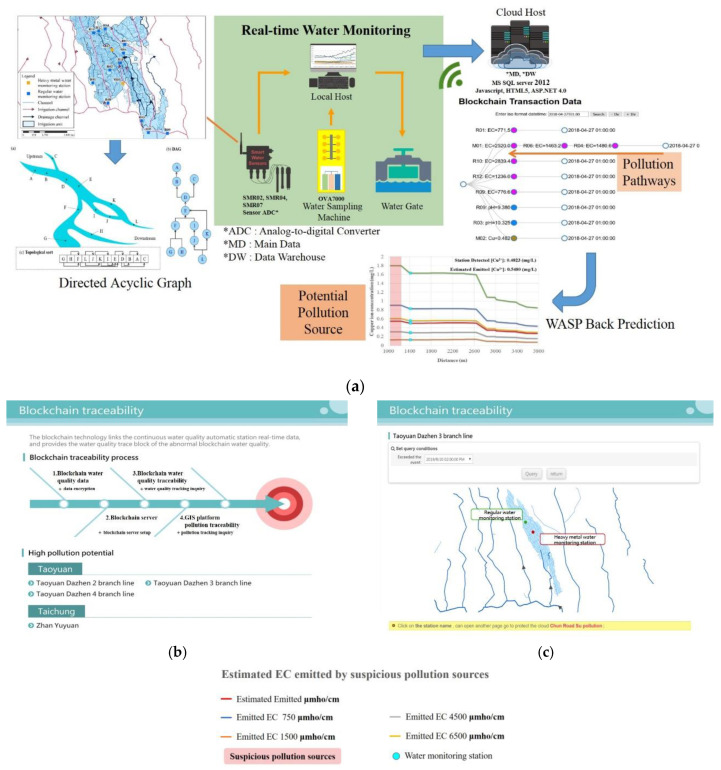
(**a**) framework of the monitoring system; (**b**) blockchain platform; (**c**) GIS platform.

**Figure 3:**
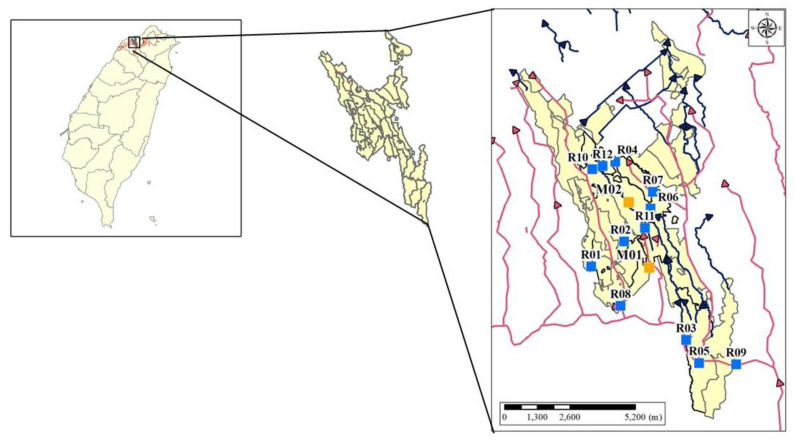
Taoyuan irrigation district study area. Note: Blue boxes represent regular water monitoring stations, and orange boxes represent heavy metal monitoring stations.

**Figure 4:**
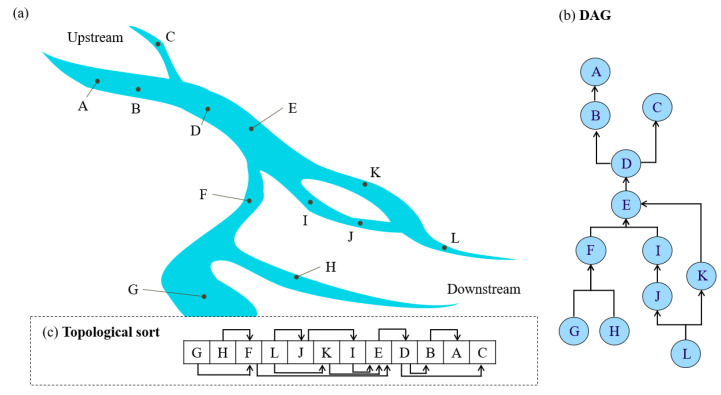
The upstream-downstream relationship between monitoring stations as a Directed Acyclic Graph (DAG) with the corresponding topological sort order (**a**) stream system, (**b**) DAG, (**c**) Sort.

This study employs a “backward pollution source tracing (BPST)” process to identify PSP. We developed a framework for pollution source tracing comprised of an IoT real-time monitoring system with sensors which form a WNS (Figure 1, Figure 2, Figure 3 and Figure 4) arranged as a directed acyclic graph (DAG); a blockchain real-time data tracing platform; a GIS spatial tracing tool; and a WASP model (Figure 1). Real-time water quality data are monitored and delivered by the IoT system, as shown in Figure S1 (see Supplementary). If the monitored water quality exceeds the regulation standard, then blockchain tracing processes are triggered, and real-time monitoring data are used to trace the pollution pathway. The GIS spatial tracing tool then uses a GIS-based irrigation channel system data to verify and spatially trace the pollution pathway. Finally, the WASP model simulates pollution concentrations along the pathway, from the sensor intake location to the pollution source. 

The concentrations of pH, temperature, Electrical Conductivity (EC), Cadmium (Cd), Copper (Cu^2+^), Zinc (Zn), Nickel (Ni), and Lead (Pb) in irrigation water have been measured by the sensors. The proportions of the above measurements greater than the regular standards are presented in Figure S1. The EC and Cu^2+^ concentrations were frequently greater than the regulation standards during our study period (Figure S1). Moreover, high EC and Cu^2+^ concentrations have also been reported in previous investigations that include the local irrigation association in the study area [6]. Accordingly, EC and copper ion (Cu^2+^) concentration data from April 2018, indicating irrigation water quality in excess of the regulation standards, were selected as real-time monitoring system examples for the proposed framework. The data were subjected to the BPST process in order to identify potential pollution sources.

The blockchain-based system was developed using G-Coin [37] The system issues a license to an address which becomes a pollution coin issuer in this study. If the water concentrations are greater than the standard allows, the pollution coin issuer “mints” a digital coin and sends the coin to the station address. Since EC and Cu^2+^ were considered the two focal pollutants for this study, two types of pollution coins were issued in the system. Transaction inputs and outputs with regard to the blockchain real-time tracing platform are shown in Figure 1.

In the blockchain transaction records, an “address” refers to one of two kinds of addresses used, namely “station address” and “collection address”, both of which either send or receive “pollution coins” at each transaction. A station address receives pollution coins and sends coins to upstream station addresses that are polluted. There is a one-to-one relationship between a monitoring station and its station address so that each monitoring station refers to a unique station address. When a monitoring station’s real-time water quality concentration data exceeds the regulation standard, a transaction between its downstream monitoring station (or coin issuer) and its station address is generated: the datum is time-stamped, thereby marking the collection address (and point in time hereafter “time point”) that all issued “pollution coins” were sent to the station address for future analysis. Collection addresses are stations that receive pollution coins and record the time point for the pollution event. That is, when a time point is triggered, thus marking a collection address, a station address is then generated. Figure 1 is a flow chart of the transaction process, which is divided into two stages: pre-processing and station operations. The system completes a full round of this uploading process for each time point generated.

In the pre-processing phase, station addresses are sorted. When a time point is triggered, pollution coins are issued, thus linking pollution coins to the station address (hereafter, “coin address”), and sha256 hash values are generated and recorded in UNIX time as private keys that correspond to the coin address. Sorting is a necessary first step since the system runs from downstream stations to upstream stations. For this reason, a DAG of the stations is made according to the upstream-downstream station relationships. The DAG is defined as follows: ‘if there is an edge (u, v) that exists in graph G, then u will be prior to v’ [38]. The vertices of the DAG are monitoring stations, and the edges are the irrigation waterways that connect two stations, with the directed edge in the opposite direction of water flow. The resulting outcome sorts downstream stations first. The WSN is also a DAG. Finally, computational complexity of the proposed approach is evaluated using Kahn’s algorithm of the big O notation [38,39]. An example of multiple pollution sources using our blockchain with a DAG approach is also provided in the Supplementary section. Details of the big O notation can be found in Avigad and Donnelly [39] and Chivers and Sleightholme [40].

### 2.1. Study Site and Experiment Design

The study area is the Taoyuan irrigation district, as shown in Figure 3. The heavy metal sensors were installed and working with the other regular sensors in the network based on all pre-investigation information and communications with the local irrigation society. Moreover, all information on the industrial plants in the study area was investigated before the installations of the sensors. Two types of sensors were installed in the study area, automatic heavy metal sensors (M) and regular sensors (R). The WSN consists of seven sensors: M02, R04, R06, R07, R09, R10, and R12 (Figure 3). The irrigation water quality standards for EC and Cu^2+^ are 750 µS/cm 25 °C and 0.2 ppm, respectively. The “regular monitoring stations” referred to in this study are PRO series monitoring stations equipped with basic water quality analyzers including pH, conductivity, ORP, dissolved oxygen, turbidity, and suspended solids. The devices connect directly via an RS485 communication interface and provide simple, reliable, cost-saving process data with remote monitoring, calibration, configuration, and diagnostics capabilities. The devices are housed in a robust IP68 proof enclosure, with a 1500 N tensile-strength Kevlar reinforced cable, and can support up to a 1.2 km digital data transmission. The transmitter is ideal for use in the water/wastewater industry.

The basic principle for measuring EC, pH, temperature, and liquid level are briefly described as follows: (1) Conductivity: two plates (cells) are placed in the sample, a potential is applied across the plates and the current is measured. Generally, the potential is in the form of a sine wave. Conductivity is determined from the voltage and current values according to Ohm’s Law:

(1) $$G=\frac{1}{R}=\frac{I}{E}$$

Since the charge on the ions in solution facilitates the conductance of electrical current, the conductivity of a solution is proportional to its ion concentration. There is a potential difference between the signal produced and measured by the sensing and reference electrodes. The theoretical potential at pH 7 is 0 mV, and the slope of the line is −59.16 mV/pH at 25 °C. This means that, in theory, the pH sensor will change its output by 59 mV for every change in a pH unit. The relationship between the potential and hydrogen ion activity in the sample is described by the Nernst equation:

(2) $$E=E_{0}+0.05916\times pH$$

where *E*_0_ is Reference potential. The temperature was measured by a Resistor Temperature Detector (RTD) sensor. In an RTD, the resistance is proportional to the temperature. RTD also requires an external current source to function properly. However, the current produces heat in a resistive element causing an error in the temperature measurements. The error is calculated by this formula:

(3) ΔT=P×S

where *T* is temperature, *P* is I squared power produced, and *S* is a degree C/mill watt. The liquid level was determined using a submersible pressure transducer by taking a continuous pressure measurement from the bottom of the tank. The pressure is proportional to the height of the liquid directly above it.

In addition to the regular monitoring stations, the Modern Water OVA 7000 was used as heavy metal monitoring stations to analyze the concentration of Cu, Pb, Cu, Zn, and Ni; based on the operations of the voltammetry principle, with a detection limit down to μg/L level, similar to ICP-MS. Furthermore, the Modern Water OVA 7000 (London, United Kingdom) accuracy avoids the interference of watercolor, turbidity, and conductivity, in order to meet the requirement of the Taiwan Council of Agriculture’s ‘Irrigation Water Standard’. This method had been approved by the U.S. Environmental Protection Agency (EPA).

The basic principle for measuring heavy metals is briefly described as follows. The anodic stripping voltammetry (ASV) method was used for measuring heavy metals. Generally, ASV is considered the most sensitive electroanalytical technique and suitable for the determination at trace levels of many metals and compounds in clinical and industrial environmental samples. In brief, the principle of ASV is based on the measurement of current signals associated with molecular properties or interfacial processes of the chemical species and is used in the detection and quantitative determination of metals or metal complexes, especially heavy metals in water.

The framework of the monitoring system with the proposed approach is shown in Figure 1. Moreover, the blockchain and GIS frameworks are also shown in Figure 1 and Figure 2.

### 2.2. Structure of the Blockchain Traceability System Used in This Study

Blockchain is a relatively new technology and the basis for many cryptocurrency transactions such as Bitcoin (https://bitcoin.org). A blockchain is a distributed ledger allowing all users to record transactions in a decentralized data log built on a peer-to-peer internet. The data in this ledger cannot be tampered with since all transactions are approved by consensus and are also encrypted. The user accesses the blockchain network and uploads information to the blockchain where it is also stored, via a node. This study developed four nodes in a blockchain network using a UTXO-based approach, where outputs of one transaction are the inputs of another information set. Each information set can be viewed as either a transaction input (TxIn) or transaction output (TxOut), thus making transactions trackable. A blockchain is a distributed ledger of transactions maintained by a network of untrusted nodes in which each block of the blockchain contains a list of transactions organized in a Merkle tree, as new blocks are added to the blockchain by users [6,16].

Information in the blockchain ledger consists of numerous transactions, so that transaction histories are disclosed. In this study, recording water quality and tracing a pollution source is similar in principle to a Bitcoin transaction. A coin representing pollution is sent from the affected downstream location to upstream locations, where the pollution events might have originated. In this way, the pollution data at one location, in relation to another location, is stored as an ordered transaction. Specifically, when a monitoring station detects pollution, the station is issued a “pollution coin”. If pollution is detected at an upstream location, coins from downstream locations are sent to the upstream monitoring station. This transaction process records the marked pathway of pollutant transportation and identifies the pollution source. In addition, based on the time when the pollution is initially detected, pollution coins are issued and sent to station addresses as recorded and time-stamped transactions.

We used the Gcoin (GCoin, 2017) blockchain in our traceability system application. The letter G in Gcoin refers to ‘global governance’ of the blockchain network. The Gcoin programming code is rewritten from Bitcoin and uses a UTXO-based blockchain network. Importantly, Gcoin can track transactions similar to the way that Mint (https://www.mint.com) can track Bitcoin transactions. Gcoin-client offers a remote procedure call (RPC) protocol to send queries. Therefore, we use Python programming language to connect Gcoin-client and to complete data upload and extraction processes.

#### 2.2.1. Uploading Water Quality Data to the Blockchain Traceability System

Figure 4 illustrates the locations of stations at both upstream and downstream areas in the upper and lower parts of the Figure, respectively, as well as the DAG of station locations. For example, though water flows from C to D, the DAG edge along this pathway is from D to C (Figure 3). While the graph illustrates the topological sort calculated with Kahn’s Algorithm [41], one should note that the topological sort depicted is not limited to only one solution (Figure 3).

After the pre-processing phase, based on the sorting order, water quality monitoring data of each station are read to verify if values have exceeded the regulation standard. If values have not exceeded the regulation standard, then the operation proceeds to the next station. If values have exceeded the regulation standard, however, then the following steps are executed: (1) Determine if there are coins in the station address ledger. If there are coins in the ledger, areas downstream of this station are polluted as well, and the pollution coins in the station address ledger can then be used directly rather than for sending additional coins to other stations; (2) If there are no coins in the station address ledger, then this station is downstream of the pollution event, and will be sent coins by the station address that was initially issued a pollution coin; (3) If stations upstream of the station address that was initially issued a pollution coin are polluted as well, then the pollution event is assumed to originate in the upstream area, and pollution coins are sent to the upstream stations; (4) If the upstream stations are not polluted, then this station address is considered the origin of the pollution event, and coins from this station address are sent to the collection address for the recording time point of the event and pollution pathways. The data uploading procedure is shown in Figure 1. For each transaction, an “OP_RETURN” output is included that records 75 bytes of information string. It is via this mechanism that the actual observed values (i.e., high EC and Cu^2+^ concentrations) and generated time points are transformed into information strings stored in the blockchain.

#### 2.2.2. Extracting Information from the Blockchain Traceability System

**Figure 5:**
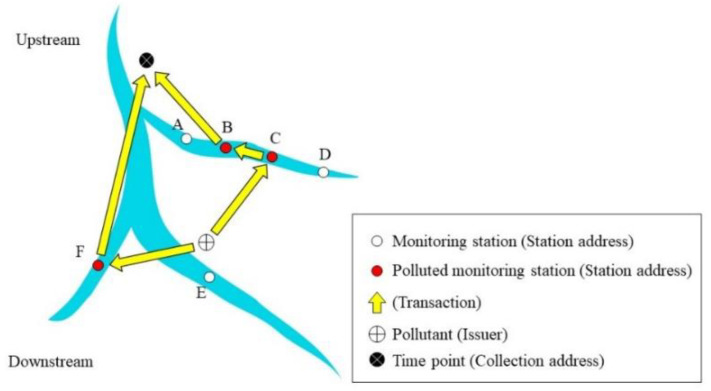
Example of the blockchain transaction record of pollution coins.

Pollution conditions at specific time points can be inspected using the following steps: (1) Identify the collection address of the time point; (2) Examine the list of pollution coins associated with the identified station address, and (3) Analyze the transaction records of these coins. An advantage of using blockchain is that it allows us to easily search the transaction history of every pollution coin. Because of this, we can rapidly identify pollution sources and affected areas. Figure 4 illustrates the mechanisms involved in a blockchain transaction record of pollution coins. In this example, our blockchain traceability system issues two coins associated with the same time point once pollution data are detected, which are then passed to the station addresses. The transaction record of the first pollution coin issued progresses from the coin issuer → Station C (station address) → Station B (station address) → collection address. The transaction record of the second pollution coin issued progresses from the same issuer → Station F (station address) → collection address. Stations B, C, and F are station addresses in the traceability system. Since the two pollution coins originate at Stations B and F, the transactions can be interpreted as follows: “two pollution events occurred at a specified time point in the upstream area of Stations B and F”. The pollution coin sent from Station B to the collection address, however, actually originated from Station C. The transactions are therefore interpreted as: “the pollution event occurred at Station B, then affected Station C which is downstream of Station B”. Furthermore, since pollution coins originating from Station C were sent from Station C to Station B; and since pollution coins originating from Station F were sent to the collection address, then “Station C and F are at the bottom of the downstream area affected by this pollution event.” The collection addresses are final addresses for the pollution events (Figure 5). Therefore, pollution pathways can be identified by the above procedure.

### 2.3. Tracking Pollution Sources with GIS

**Figure 6:**
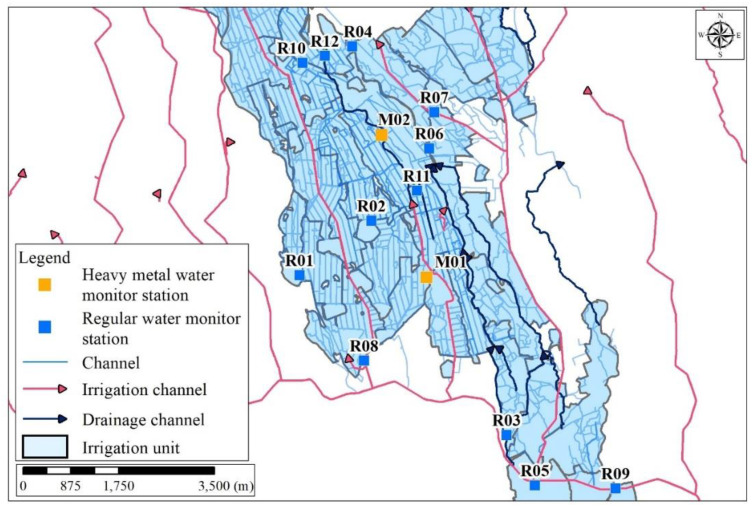
Spatial distributions and irrigation flow with monitoring stations in the study area. Note: R is a regular water monitoring station; M is a heavy metal monitoring station.

In the Taoyuan irrigation district study area, the local water utility administration is the responsible authority for irrigation water resources management. Due to a limited number of sensors, after using blockchain to trace monitoring station transactions, we mapped the Taoyuan irrigation district irrigation unit with GIS as a first step to track the pollution source with GIS. Upstream and downstream relationships were determined by the direction of flow through the irrigation channels, based on the DAG analysis. Mapping the study area’s local water utility administrative area with GIS allowed us to identify nearby administrative areas along the irrigation canal, as well as relevant irrigation and farmland information, such as drainages and other hydraulic structures (Figure 6). Second, we mapped factories that were identified as likely pollution sources according to information derived from our blockchain traceability system. Third, we mapped additional spatial layers of information on water monitoring stations, soil survey data, and factory characteristics to conduct further analysis of the industrial factories identified as likely pollution sources and their related polluted areas. Lastly, we compared the type of pollution detected by our blockchain traceability system, with the factory characteristics of the industrial factories screened in the last step, to eliminate irrelevant industrial factories.

**Figure 7:**
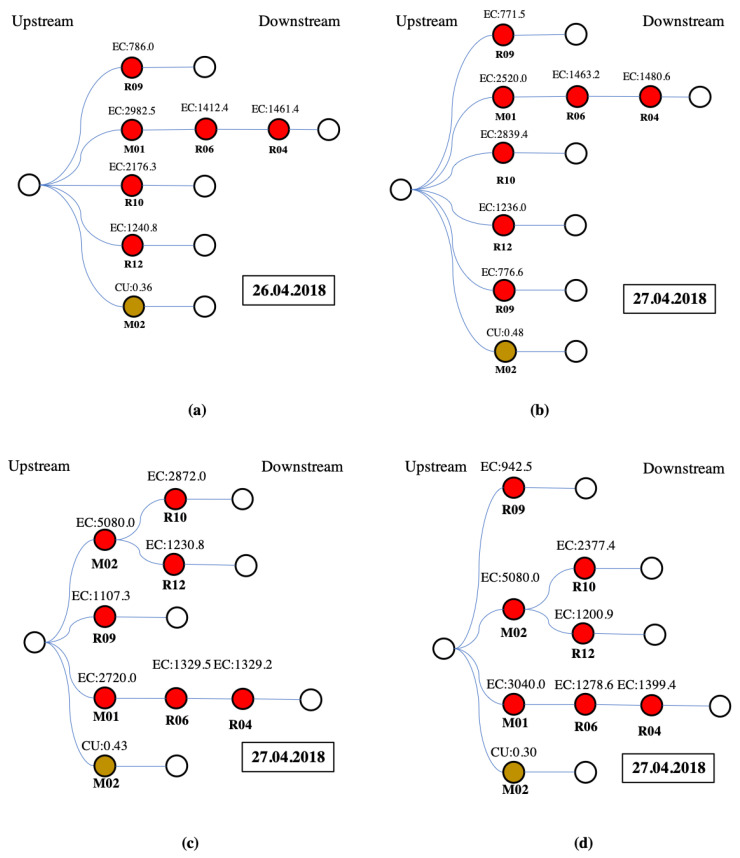
Procedure trees for four monitoring stations with detected EC and Cu concentrations exceeding the regulation standard. Note: Similar data for the remaining six scenarios are not shown. (**a**) Case 1 (Type I); (**b**) Case 3 (Type II); (**c**) Case 4 (Type III); (**d**) Case 5 (Type IV). Red circles represent Electrical Conductivity (EC); Brown is Copper (Cu^2+^); R is a regular water monitoring station; M is a heavy metal monitoring station; the Date of the transaction is indicated in the outlined box as day–month–year.

**Figure 8:**
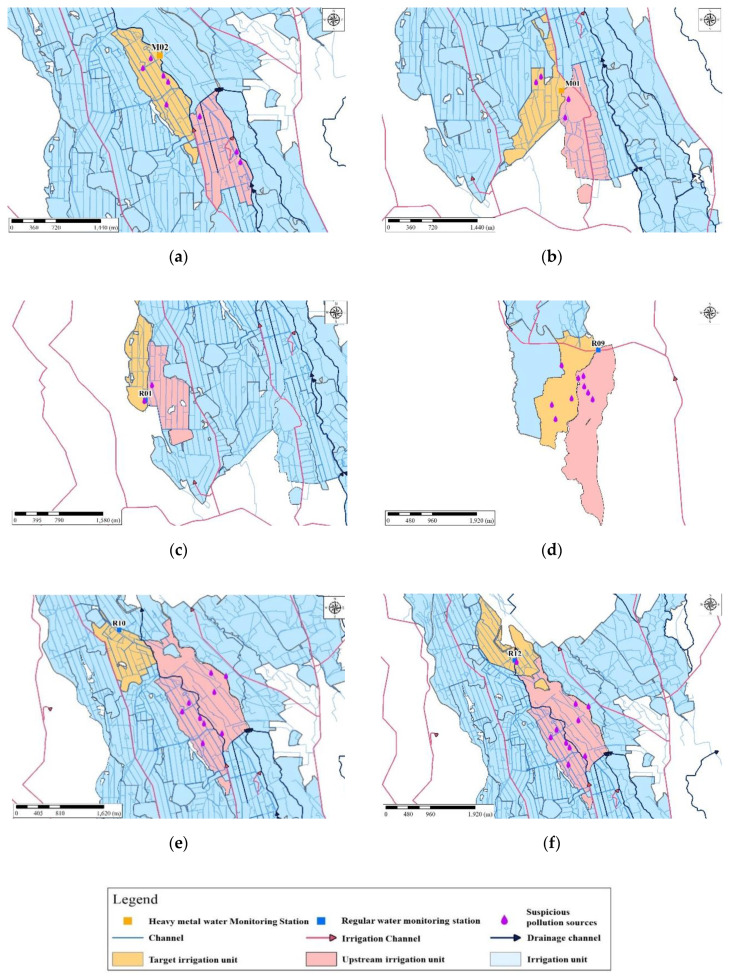
Pollution pathways of upstream and downstream irrigation units for (**a**) Case 1 (Type I); (**b**) Case 3 (Type I); (**c**) Case 4 (Type II); (**d**) Case 5 (Type III); (**e**) Case 6 (Type IV); (**f**) Case 8 (Type V). Note: Red is the polluted upstream irrigation unit; R is the regular water monitoring station; M is the heavy metal monitoring station.

In this study, we integrated a blockchain traceability system with GIS to track pollution sources in the San–Kuai–Tsuo irrigation channel, the 3rd branch of the Taoyuan Canal within the Taoyuan irrigation district study area (Figure 6). Figure 7 shows the pollution pathways using the traceability system. Using a drainage tracking function on the internet platform (Figure 1), we designated impacted areas (orange color) and upstream areas (purple color) based on irrigation units (Figure 8). Figure S2 shows the DAG of the study.

### 2.4. Simulation of Wastewater Discharge Quality

The Water Quality Analysis Simulation Program model is a mass balance equation developed by the US EPA [36].for dissolved constituents of the water body, which accounts for all the material entering and leaving through direct and diffuse loading; advective and dispersive transport; and physical, chemical, and biological transformations [36]. We used WASP (US EPA) Version 7.3 to simulate EC and Cu^2+^ concentration in wastewater discharge from industrial factories within the Taoyuan irrigation district study area. The data used for the WASP simulation were collected by field investigation and water quality monitoring stations. We used Manning’s equation to calculate the flow rate in the irrigation canal, and incorporated Cu^2+^ concentrations that were measured in water and sediment, to conduct the simulation. The partial differential equation with the Runge–Kutta method was used for the simulation:

Water quality control equation [36]:

(4) $$\partial C/\partial t+\partial UC/\partial x-\partial \left(E_{x}\partial C/\partial x\right)/\partial x-\partial \left(E_{Z}\partial C/\partial z\right)/\partial z=S_{L}+S_{k}$$

Sediment control equation:

(5) $$\partial C/\partial t-\partial \left(E_{z}\partial C/\partial z\right)/\partial z=S_{k}$$

where *U* is flow velocity of the *x*-direction (m/s); *C* is contaminant concentration (g/m^3^); *E_x_* and *E_Z_* are *x*- and *z*-direction dispersion coefficients (m^2^/s); *S_L_* is external load (g/m^3^/s); and *S_K_* is source and sink (g/m^3^/s).

The San–Kuai–Tsuo irrigation channel was divided into 32 segments (Table S7). The length of the channel is 3838 meters, with a slope of 0.002. The WASP model irrigation channel parameters for model calibration were established based on field survey results (see Supplementary). Heavy metals emitted from likely sources in the upstream area are traced by utilizing the heavy metal monitoring station data on EC concentration, Cu concentration, and water level. Model parameters are listed in Table S1 (see Supplementary). Given real-time measurements of EC and Cu^2+^ at the identified water intake (downstream), the WASP model simulates the EC and Cu^2+^ concentrations at all segments along the above irrigation channel by using an iteration procedure. The iteration procedures are not stopped until the simulated concentrations of EC and Cu^2+^ at the intake of irrigation water are close to those of the measured concentrations. The Mean Absolute Percent Error (MAPE Equation (S1) see Supplementary) values of model validations for EC and Cu^2+^ were 9.58% and 3.74%, respectively. Moreover, the R^2^ values of model validations for EC and Cu^2+^ were 0.9986 and 0.9682, respectively (Figure S2 in Supplementary). The MAPE and R^2^ values show that the simulation model has the ability to simulate EC and Cu^2+^ concentrations.

### 2.5. Computational Complexity

In this study, we assumed that the total monitor station number is V (R + M, R: number of regular water monitor stations, M: number of heavy metal water monitor stations). The total number of edges (which connect the stations) is E. Additionally, assume that the DAG is using an adjacency list in which each node stores the outgoing edges. By using Kahn’s algorithm:

Step 1: Make an AdjList with the current in-degree of each node and initialize the count of visited nodes as 0.

Step 2: Make a queue of the set of nodes with in-degree 0 (Enqueue operation).

Step 3: Remove a vertex from the queue (Dequeue operation) and then:

Increment count of visited nodes by 1.

Reduce in-degree by 1 for all nodes adjacent to it.

If the in-degree of an adjacent node is reduced to zero, then add it to the queue.

Step 4: Repeat Step 3 until the queue is empty.

Step 5: If the count of visited nodes is not equal to the number of nodes in the graph, then the topological sort is not possible for the given graph.

In addition, pollution conditions at specific time points can be inspected using the following steps: (1) Identify the collection address of the time point; (2) Examine the list of pollution coins associated with the identified station address, and (3) Analyze the transaction records of these coins.

The Algorithm 1 for the above pollution analysis algorithm is as follows.
**Algorithm1:** Pollution Analysis Algorithm.Pollution Analysis { Identify the collection address of the time point //step 1   Sc = set of all pollution coins;   For each c ∈ Sc {//step2 & step 3   Generate pollution pathway Pc for c’s transition record.   Analyze Pc to determine which is the pollution source    //the pollution source is at the upstream of the sources in Pc,   // the other is pollution path.   } }

## 3. Results

### 3.1. Uploading Water Quality Data to the Blockchain Traceability System

Figure S3. Shows the DAG of the stream system. The EC and Cu^2+^ results for 14 water monitoring stations are categorized as either “exceeding” or “non-exceeding” regulation standards, for which there are 30 combinations (Tables S8 and S9). In this study, there were ten instances (Tables S10–S19) in which water monitoring stations were categorized as exceeding regulation standards for EC and Cu^2+^ concentrations (Tables S8 and S9). The water quality data upload procedures for these ten cases are shown in Tables S10–S19 (Supplementary).

The blockchain results for the ten cases of pollution pathways (Tables S10–S19) are summarized as the following four types of pollution pathways: Type I are cases 1–2 (based on Tables S10 and S11); Type II is case 3 (based on Table S12); Type III is case 4 (based on Table S13); and Type IV are cases 5–10 (based on Tables S14–S19). For the above four pathway types for which pollution coins were issued, Figure 7 shows the pollution pathways based on the blockchain transaction data as procedure trees. Blockchain transaction data in Figure 7 can be read from right to left--downstream stations on the right-hand side of the procedure tree are sent to upstream stations on the left-hand side of the procedure tree, along various pathways comprised of irrigation channels (Figure 7). In Figure 7, water monitoring stations are represented as circles, and color-filled circles represent water monitoring stations that detected irrigation water pollution concentrations exceeding the regulation standard. For instance, since the EC concentration detected at Station R04 was greater than the regulation standard, Station R06 received pollution coins from Station R04 in the blockchain traceability system (Figure 7). At the same time, Station M02 received pollution coins from Stations R10 and R12, since the EC concentrations detected at Stations R10 and R12 exceeded the regulation standard. Moreover, Station R09 also issued a pollution coin since the detected EC concentration exceeded the regulation standard, though no pollution coins were sent to or from Station R09 (Figure 7). These transaction “data pathways” cannot be tampered with in the blockchain traceability system.

With respect to heavy metal results, during our study period, there were ten cases in which Cu^2+^ concentrations exceeded regulation standards and were recorded as having concentrations of 0.358 ppm, 0.595 ppm, 0.482 ppm, 0.429 ppm, 0.299 ppm, 0.271 ppm, 0.209 ppm, 0.226 ppm, 0.393 ppm, and 0.316 ppm, respectively. Detailed data uploading procedures for each case can be found in the Supplementary Materials (Tables S10–S19). Since only two heavy metal monitoring stations were installed in the study area, heavy metal results from these two water monitoring stations were categorized as either “exceeding” or “non-exceeding,” for which there are two combinations representing two pathway types (Table S9 and Figure 7). For each combination, Type II (Table S9) occurs most often. Occurrence times for each combination are presented in Figure 7. Unknown pollution sources were detected in the upstream area of the study area using downstream monitoring data. For Type II, only Station M02 exceeded the Cu^2+^ concentration regulation standard. According to the operation order (i.e., topological sort order), the pollution progresses along the following pathway: R04, R06, R07, R10, R12, and M02 (Figure 7). 

In this study, an example of multiple pollution sources was provided in the Supplementary section. Figure S4 shows the network of the example with two types of pollution sources, and consists of 11 heavy metal sensors (see Supplementary). Figure S4 also shows the upstream-downstream relationships between monitoring stations with the corresponding topological sort order. Using our approach (Figure 1), pollution progresses along the following pathways presented for the multiple pollution sources displayed in Figure S5 (see Supplementary). The pollution type #1 has a simple pathway through Station D that leads to the pollution source, but the pollution type #2 has three pathways that lead to pollution sources (Figure S5). 

### 3.2. Mapping Industrial Factories Identified as Likely Pollution Sources with GIS

After mapping local water utility administrative areas with GIS, we mapped pollution sources identified by the blockchain traceability system with real-time monitoring data. To reflect the current irrigation system delineated with GIS, upstream irrigation units are depicted in red (Figure 8). Since we used a limited number of wireless sensors to monitor irrigation water intake, we pinpointed the polluted irrigation units located within the administrative area instead, and marked these irrigation units with violet-colored water drop symbols in GIS (Figure 8).

For instance, Station M02 received pollution coins from Stations R10 and R12 in the blockchain traceability system (Figure 8a) when the EC concentrations at Stations R10 and R12 exceeded the regulation standard. We then identified highly likely sources of pollution at target irrigation units within the Taoyuan irrigation district, by narrowing down the likely sources along the pollution pathway (i.e., from downstream water monitoring stations to the originating stations); and then to the industrial factories in this vicinity (Figure 8a). Using GIS tools, upstream irrigation units and pollution intake locations are thus identified (Figure 8a). Moreover, we can further identify the likely pollution sources—specific industrial factories—from various GIS graphic layers with aerial photography and farmland monitoring data. The GIS mapped results for the ten cases are summarized in Figure 8 as five types of spatial pathways. That is, these ten cases are the same spatial pathway types depicted in Figure 7. Specifically, Type I are cases 1–3 (based on Tables S10–S12); Type II is case 4 (based on Table S13); Type III are cases 5, 7, 9, and 10 (based on Tables S14, S16, S18 and S19); Type IV is case 6 (based on Table S15); and Type V is case 8 (based on Table S17). 

### 3.3. Simulation of Wastewater Discharge Quality

**Figure 9:**
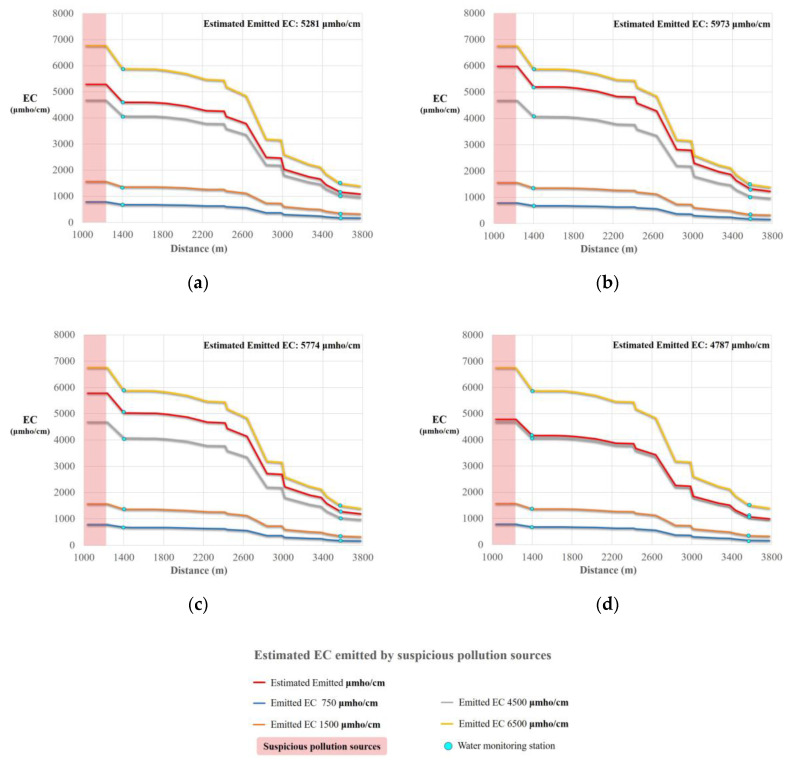
Reverse prediction of EC concentrations along the channel for each case at heavy metal monitoring station M02 (**a**) 5080 µmho/cm; (**b**) 5746 µmho/cm; (**c**) 5560 µmho/cm; (**d**) 4610 µmho/cm.

**Figure 10:**
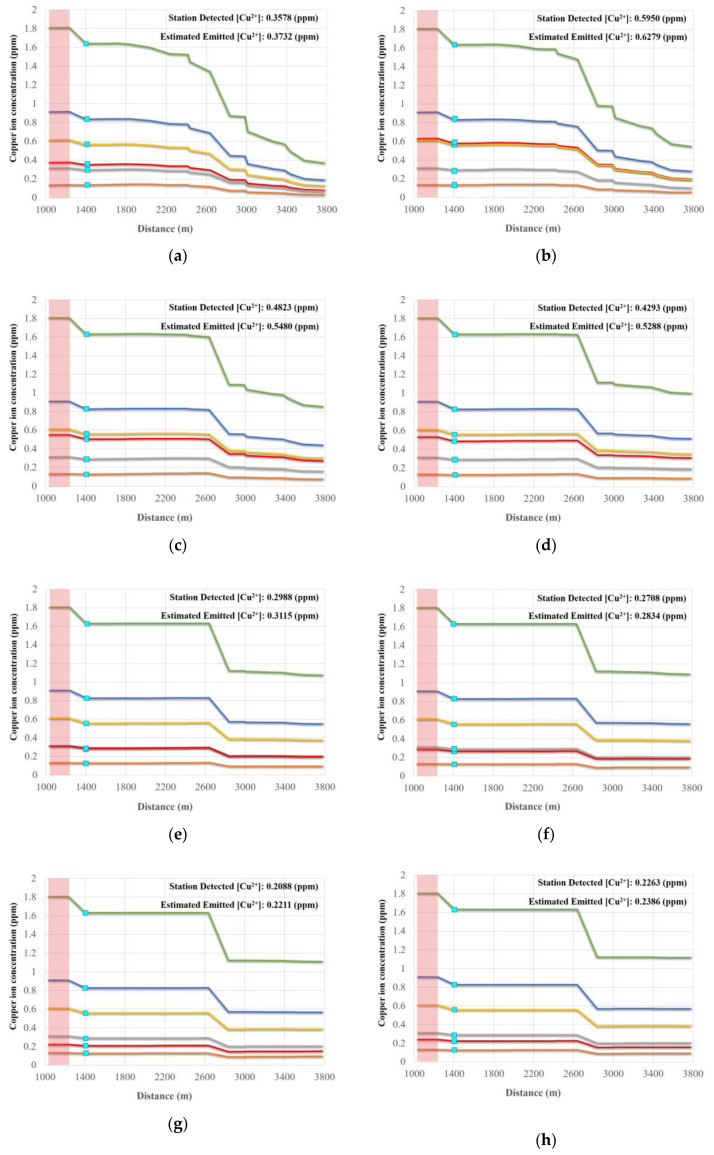

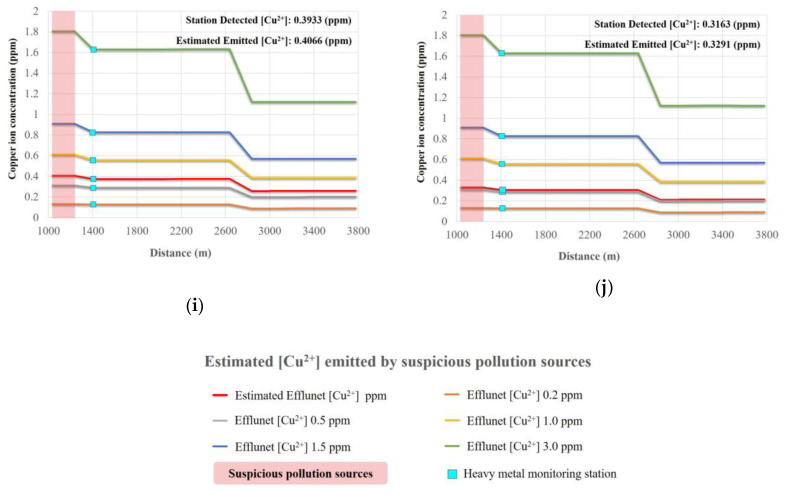
Reverse prediction of Cu^2+^ concentrations along the channel for each case at heavy metal monitoring station M02 (**a**) 0.358 ppm, (**b**) 0.595 ppm, (**c**) 0.482 ppm, (**d**) 0.429 ppm, (**e**) 0.299 ppm, (**f**) 0.271 ppm, (**g**) 0.209 ppm, (**h**) 0.226 ppm, (**i**) 0.393 ppm, and (**j**) 0.316 ppm.

A water monitoring station was located 200 m downstream from one of the likely pollution sources. Data collected from this station was used for simulating wastewater discharge quality. During the field investigation, we found that the shape of the irrigation channel was an inverted trapezoid, with a total length and head loss of 3838 m and 19.89 m, respectively. We input all required measured data (e.g., flow and water depth) from the ten abovementioned cases into WASP, to simulate wastewater discharge quality. Figure 8 and Figure 9 show the WASP model-simulated trends for EC and Cu^2+^ concentrations. The simulations show that increasing concentration trends are potentially caused by a likely pollution source (Figure 9 and Figure 10), since concentrations rapidly jump in all cases where the irrigation water quality exceeds regulation standards for EC and Cu^2+^.

### 3.4. Computational Complexity

In this study, we conducted a simple complexity analysis for our pollution analysis algorithm with the following. By using Kahn’s algorithm, the time complexity of sorting the DAG is O(V + E). Step 1 takes O(V) time to identify the collection address of the time point. We need O(V × E) for executing Step 2 and Step 3 of our approach (Figure 1), i.e., there are V nodes and, for each node, we need to spend O(E) for examining the pollution pathway and identify the pollution source. Therefore, the total time complexity of our pollution analysis algorithm is O(V × E). For the case of Figure 3, there are 12 monitor stations in the stream system. Since there are 12 nodes and 12 edges in the DAG, the computational complexity is O(12 × 12). If there are n polluted stations, the computation complexity is O(V × E + n).

**Figure 11:**
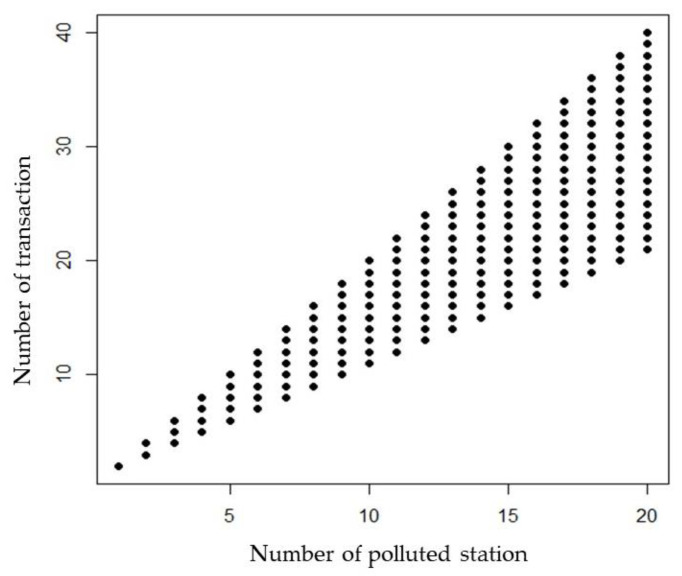
Big O notation analysis of the relationship in the number of Blockchain transactions and the number of polluted stations.

In the real case, computational complexity was done and discussed for the DAG, Blockchain transaction and WASP modeling by using the big O notation to analyze time complexity. The total time complexity of our pollution analysis algorithm is O(14 × 10 + n) in the study case. The relationship between Blockchain transactions and the number of polluted stations (sensors with concentrations greater than the regulation standards) show increasing linear trends (Figure 11). Once n stations are polluted, the least number of transactions is n + 1, and the maximum number of transactions is 2n (Figure S3). In the two pollution sources designed case, the total computational complexity is O(11 × 10 + 7) since the number of polluted stations is 7. Figure 11 indicates that the number of transactions strongly relies on the number of sensors with concentrations greater than the regulation standards. Since the WASP model is a deterministic model, the computation is the model computation. The computational times of the model increased from 4, 137, 142, and 180 min for the cases with numbers of segments increased from 32, 93, and 128, respectively.

## 4. Discussion

### 4.1. Uploading Water Quality Data to the Blockchain Traceability System

This study is the first to utilize blockchain technology with a WSN in a DAG configuration to identify pollution pathways and to trace pollution sources based on real-time irrigation water quality data. Pollution tracing in this way was possible only with blockchain technology’s characteristic immutability, traceable transactions, and transparency [6,10]. As a result, the real-time pollution data used in this study cannot be altered. Additionally, pollution dispersion can be identified with this study’s use of pollution coins, issued by the blockchain traceability system, so that transactions can be traced through the irrigation systems with a DAG to identify polluted pathways in our study area. Although the issued pollution coins were not actual commercially-traded currency, the concept of pollution coins can be further utilized in water quality management schemes.

Our approach differs from traditional WSN uses, in that the proposed blockchain-based approach uses a WSN as a DAG to sort the network, based on the spatial relationships between water monitoring stations in the study area. While our traditional client-service model was developed based on a time series of the water monitoring data from a station suited for Structured Query Language (SQL) protocols, the water quality measurement data that exceed regulatory standards have been recorded in this study’s blockchain and should not be altered since the data are verifiable evidence that can be used for further actions. Moreover, blockchain encryption prevents any data manipulation of the monitoring data in this study, even when water quality data and events are transferred among monitoring stations. That is, the historical and real-time events used in this study were transferred as hash values (i.e., digital fingerprints) because the data are managed by a peer-to-peer network for inter-node communication, making it immutable. The recorded blockchain events in this study are transparent, traceable, and secured data that can then be utilized for water quality management. Future studies employing our blockchain tracing procedure should include additional sensors set at suitable upstream channels within the irrigation unit to overcome any issues due to a lack of upstream water monitoring station data if there are no budgetary issues. On the other hand, the proposed approach can be a tool to reduce the number of sensors and cost for the installation of too many sensors. Our results in the example case also indicated that the proposed approach could be utilized for multiple types of pollution. 

### 4.2. Mapping Industrial Factories Identified as Likely Pollution Sources with GIS

Unlike other relevant studies using GIS to identify potential pollution or risk zones [29,32,33], this study developed a blockchain-based GIS system for real-time identification of polluted upstream irrigation units, via a WSN within irrigation channels in the study area. Due to a limited number of sensors in the study area, the upstream to downstream irrigation units were fixed in a basic spatial relationship, using GIS tools (Figure 8). Our method, however, can be applied to complex networks as a cost-effective method for determining sensors within a network. The upstream unit used in this study, for the cases in which EC and Cu^2+^ concentrations exceeded the regulation standard, is a real-world upstream unit. Although copper is essential to the growth of plants, soil copper concentrations in excess (i.e., beyond a threshold of 400 mg/kg for rice) have inhibitory effects on crop growth and development [42], suggesting a need for real-time water pollution monitoring. GIS techniques used in this study differ from other blockchain WSN techniques used to spatially identify polluted irrigation units reported by [31,43]. Our use of a GIS tracking procedure, initiated directly after the blockchain process, effectively identifies polluted upstream irrigation units and irrigation water intake locations in the study area for real-time water quality modeling. Furthermore, using GIS tools can reduce sensor installation, sensor maintenance, and water monitoring station costs if operating under a limited budget.

### 4.3. Simulation of Water Quality for Real-Time Pollution Source Tracking 

Water quality models or indices that are GIS-based have been widely developed to assess water quality and to identify potential pollution source risk and risk areas [44,45]. In the last two decades, the WASP has been successfully used to simulate the arrival time of pollutants from various locations and concentrations after water pollution accidents, as dynamic GIS layers [32]. This study utilized the WASP model for successful reverse prediction of EC and Cu^2+^ concentrations with real-time water quality monitoring data and real-time pollution source tracing via a WSN in the study area. In order to trace pollution sources in real-time, our proposed approach auto-initiates once EC and Cu^2+^ concentrations exceed regulation standard allowances. Unlike studies that only use a WSN approach [46,47], this study used blockchain technology transaction functions and a DAG. Although we readily concede that our model traced a pollution source that was within relatively proximity to a monitoring station (within a 200 m radius), increasing the sensor amount and performing model calibrations based on a longitudinal data set will improve traceability of large-scale unknown pollution sources when using the proposed framework. However, overcoming potential modeling challenges may require clarification on the flow directions between upstream and downstream drainage areas, water monitoring station locations, and irrigation facility and channel information. 

### 4.4. Computational Complexity

As far as we know, our study is the first study using a DAG and Blockchain to identify PSP. Complexity evaluation of an algorithm is essential for algorithm design [48]. Our computation analysis indicates that the computational time of the Blockchain transactions, and the number of sensors with high concentrations can be estimated. Moreover, the maximum transaction number, 2n, is not influenced by the upstream-downstream relationship once all sensors detect concentrations greater than the regulation standards. The computational time of DAG relies on the number of sensors and edges of irrigation networks. The least transaction number, n + 1, occurs once all pollution is detected by sensors along the same irrigation channel. The computational time of the DAG is essential for our proposed approach. Once the number of pollution types detected by stations increases the computational time will increase additionally. However, a DAG-based approach has been recently discussed as a way to revolutionize the blockchain technology [49,50]. The advantage of our study is that a DAG was utilized to order the transaction and realize the computational time.

## 5. Conclusions

Real-time identification of PSP is essential for ensuring irrigation water quality. While blockchain’s distributed ledger technology allows all users to record transactions in a decentralized data log built on a peer-to-peer internet, a WSN provides real-time remote monitoring data for high-quality production and processing systems with various sensors that are applicable in different fields of study. This study’s novel framework uses blockchain-based technology, a WSN in a DAG configuration, and GIS techniques to trace pollution pathways from irrigation water intake data. We then simulated irrigation water quality using a WASP model to successfully identify PSP for ten case studies. Since blockchain encrypts data and prevents data manipulation as water quality data are transferred among monitoring stations, the secured data used in this study can be useful for further real-world water quality management. Pollution sources and pathways of irrigation units can be targeted for further field investigation using the proposed blockchain traceability system with GIS tools. Once a monitored polluted irrigation unit is mapped with GIS following the blockchain tracing procedure, a WASP water quality simulation provides information for real-time identification of highly likely pollution sources that engage in illegal wastewater discharge. The computational complexity of the Blockchain transactions shows a linearly increasing relationship with the number of polluted stations. The proposed framework can be utilized in complex water quality monitoring networks with multiple pollution sources to identify PSP. The computational complexity of the transactions in the proposed framework should be evaluated. 

## Supplementary Materials

The following are available online at https://www.mdpi.com/1424-8220/20/13/3634/s1, Figures S1 and S2 show the proportions of (a) Electrical Conductivity (EC); (b) Cadmium (Cd); (c) Copper (Cu^2+^); (e) Lead (Pb); (e) Nickel (Ni); (d) Zinc (Zn) greater than the standards and the validations of the water quality model; Tables S1–S7 show parameters of the WASP model; Tables S8 and S9 show groups with combinations of EC and Cu^2+^ concentrations exceeding the regulation standards during April 2018. Tables S10–S19 show data upload processes through the pollution pathways using transactions.
