# Supplementary material for: Real-Time Identification of Irrigation Water Pollution Sources and Pathways with a Wireless Sensor Network and Blockchain Framework
Source: Sensors (Basel). 2020 Jun 28;20(13):3634. doi: 10.3390/s20133634 (PMC7374519; doi:10.3390/s20133634)
Supplement: Supplementary file 1 [file sensors-20-03634-s001.pdf]

Supplementary
S1. Water quality information

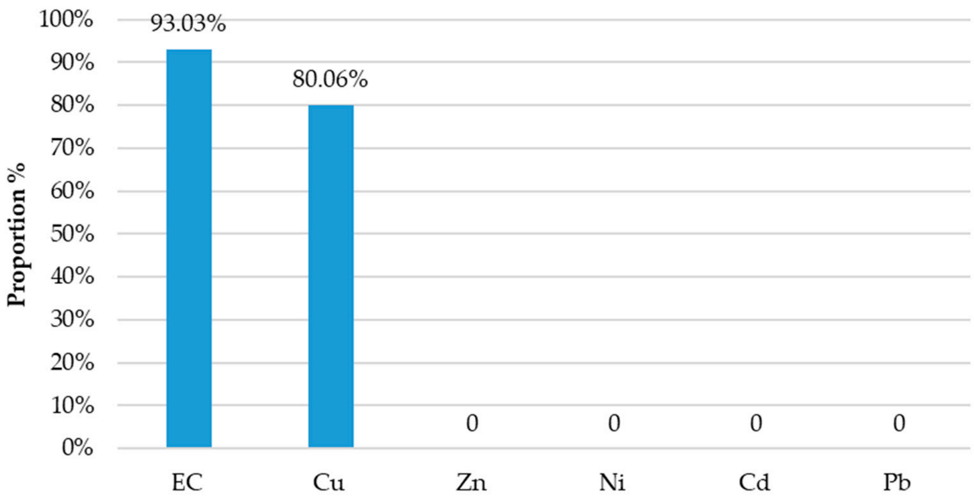

Figure S1 proportions of Electrical Conductivity (EC), Cadmium (Cd), Copper (Cu2+), Lead (Pb), Nickel (Ni), Zinc (Zn) greater than the standards.

S2. WASP model parameters

The model parameters and segment information are listed in Tables S1-S7. The parameters and segments are set and reported in the same study area based on real measurements (Tables S1-S7) (Taiwan Council of Agriculture, 2016) [1]. The calibrations are based on the Taiwan Council of Agriculture (2016) report on modeling procedures of the WASP (https://www.epa.gov/ceam/modeling-products-assess-exposures). All procedures for data and mreasurements are presented in the user's guide of the model [2]. In this study, the EC and Cu^{2+} concentrations were simulated at each segment based on the parameters and data from the report (Tables S1-S7). Figure S2 shows the model validation for the EC and Cu^{2+}. The R2 calues are 0.9986 and 0.9682 for EC and Cu^{2+}, respectively. The Mean Absolute Percent Error (MPAE) equation is as follows. The value of MPAE is less than 10% that indicates high accurate simulation [3].

$$\text{MAPE} = {(\sum|measurements - simulated\ value|/|\ measurements\ |)100}/{n} \quad \text{(S1)}$$

Note: n=number of segments.

Table S1. Model data set and paramters

Data set	Parameters
**Model type**	Simple Toxicant
**Start date**	1/1/2017
**Start time**	00:00
**End Date**	2/1/2017
**End time**	00:00
**Hydrodynamics**	1-D Network Kinematic wave
**Solution Technique**	Runga-Kutta
**Bed volumes**	Static
**Fraction of max time step**	0.01
**Max time step**	0
**Min time step**	0.0001

Taiwan Council of Agriculture (2016)

Table S2. Segment information

Segments	Value
**Segments**	as **Table S7** Segment information
**Initial Concentrations**	
Toxicant: Seg1~6	200
Silts and fines: Seg1~6(20); Seg7~12	10000
**Fraction dissolved (Toxicant, Silts and fines, Sand, Organic solids, Tracer 1&2)**	1

Taiwan Council of Agriculture (2016)

Table S3. Paramter scale factor

Parameter data	Scale factor
**Partition Coefficient to Silts and fines(L/kg)**	1258.9254

Taiwan Council of Agriculture (2016)

Tables S4. Constant parameters

Constants data	Value	Min	Max
**Constant group: Solids**			
Biotic Solids Production Rate (g/m3-day)	2	0	5

Organic Matter Dissolution Rate Constant (1/day)	0.004	0	0.5
Organic Solids Fraction Ash (Ash/DW)	0.1	0	1

Taiwan Council of Agriculture (2016)

Table S5. Volumes

Loads	CMS	kg/day
**Toxicant:**		
**Segment** 12	0.013854	1.46736
**Segment** 13	0.0807	9.01104

Taiwan Council of Agriculture (2016)

Tables S6. Flow

Flow	Value
**Surface water**	V
**Solids 1**	V
**Time/value pairs for Surface Water, main**	0.053

Taiwan Council of Agriculture (2016)

Table S7. Segment information

Segment	Volume	Depth (m)	Segment Type	Length (m)	Width (m)	Slope	Bottom Roughness	Velocity (m/s)	Volume
1W	6.463	0.214	surface water	38	7.064	0.0069	0.032	0.290	2684.404
2W	30.108	0.214	surface water	200	7.064	0.0069	0.032	0.210	14128.440
3W	15.957	0.214	surface water	106	7.064	0.0069	0.032	0.199	7488.073
4W	9.183	0.214	surface water	61	7.064	0.0069	0.032	0.199	4309.174
5W	4.968	0.214	surface water	33	7.064	0.0069	0.032	0.199	2331.193
6W	6.022	0.214	surface water	40	7.064	0.0069	0.032	0.199	2825.688
7W	16.596	0.215	surface water	110	7.065	0.0068	0.032	0.310	7770.950
8W	7.560	0.215	surface water	50	7.065	0.0068	0.032	0.270	3532.250
9W	19.202	0.215	surface water	127	7.065	0.0068	0.032	0.198	8971.915
10W	11.038	0.215	surface water	73	7.065	0.0068	0.032	0.198	5157.085
11W	6.804	0.215	surface water	45	7.065	0.0068	0.032	0.198	3179.025
12W	24.423	0.233	surface water	155	7.070	0.0052	0.032	0.385	10958.360
13W	52.856	0.515	surface water	200	7.155	0.0052	0.032	0.500	14309.120
14W	60.527	0.515	surface water	166	7.155	0.0052	0.032	0.309	11876.569

15W	28.803	0.584	surface water	74	7.175	0.0029	0.032	0.360	5309.618
16W	66.213	0.584	surface water	160	7.175	0.0029	0.032	0.250	11480.256
17W	35.176	0.584	surface water	85	7.175	0.0029	0.032	0.250	6098.886
18W	50.239	0.383	surface water	115	12.038	0.0022	0.025	0.212	13844.045
19W	91.980	0.383	surface water	200	12.038	0.0022	0.025	0.212	24076.600
20W	91.980	0.383	surface water	200	12.038	0.0022	0.025	0.212	24076.600
21W	83.242	0.383	surface water	181	12.038	0.0022	0.025	0.200	21789.322
22W	8.738	0.383	surface water	19	12.038	0.0022	0.025	0.212	2287.277
23W	76.620	0.255	surface water	200	12.026	0.0085	0.025	0.319	24051.000
24W	61.260	0.255	surface water	200	12.026	0.0085	0.025	0.319	24051.000
25W	44.795	0.242	surface water	150	12.024	0.0085	0.025	0.308	18036.301
26W	7.856	0.242	surface water	27	12.024	0.0085	0.025	0.308	3246.534
27W	88.200	0.338	surface water	253	12.034	0.0028	0.025	0.221	30445.514
28W	55.253	0.475	surface water	113	12.048	0.0009	0.025	0.183	13613.675
29W	24.870	0.251	surface water	57	12.025	0.0063	0.025	0.271	6854.307
30W	42.136	0.251	surface water	140	12.025	0.0063	0.025	0.271	16835.141
31W	57.866	0.231	surface water	200	12.023	0.0067	0.025	0.215	24046.199
32W	16.661	0.231	surface water	60	12.023	0.0067	0.025	0.380	7213.860

Taiwan Council of Agriculture (2016)

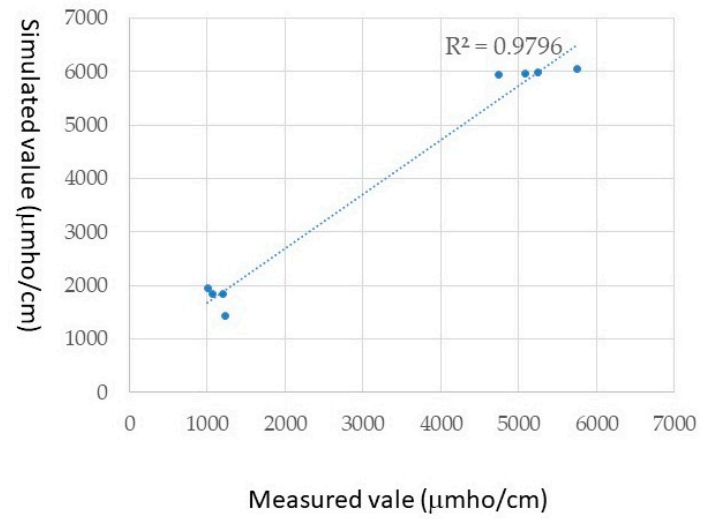

(**a**)

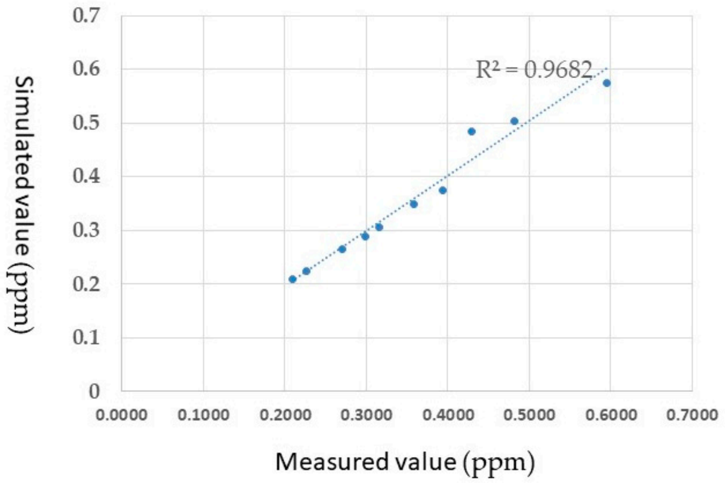

(**b**)

Figure S2. Measure values v.s. Simulated valued (a) Electrical Conductivity (EC); (b) Copper (Cu^{2+}).

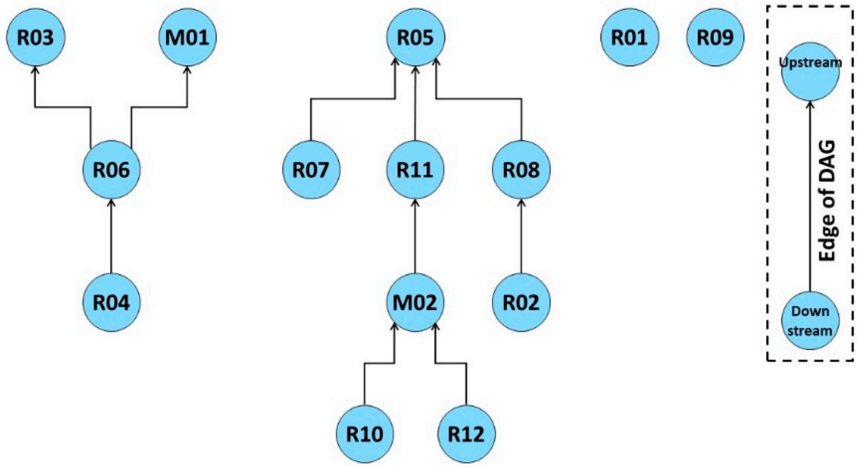

Figure S3. The upstream-downstream relationship between monitoring stations as a Directed Acyclic Graph (DAG) (modified from Huang et al., 2019 [4]).

S3. A design case of multiple pollutions

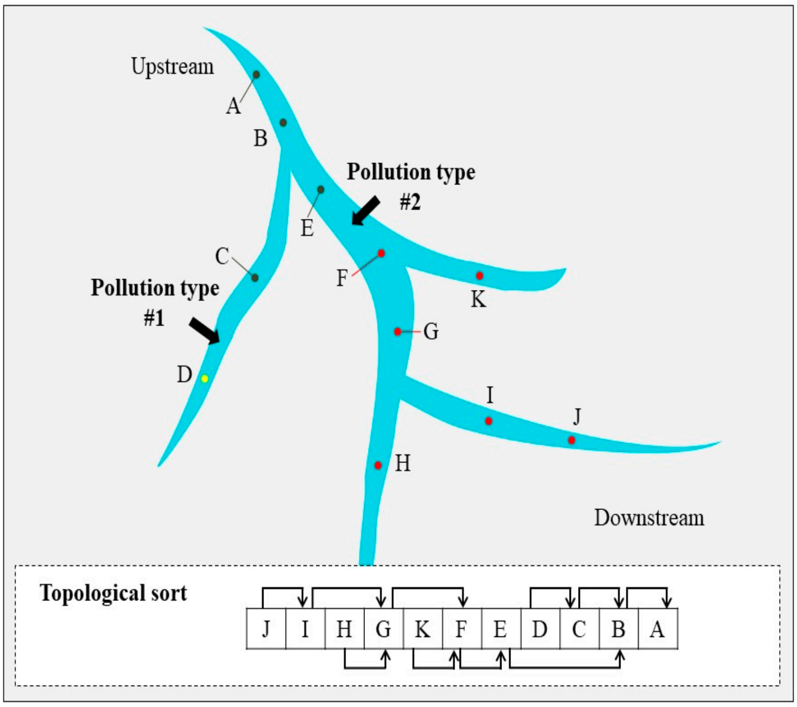

Figure S4. The upstream-downstream relationship between monitoring stations and topolpgical sort.

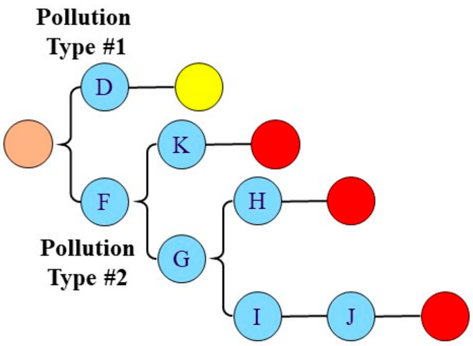

Figure S5. Procedure trees for four monitoring stations with detected EC and Cu concentrations exceeding the regulation standards.

S4. Tables

Table S8. Groups with combinations of EC concentrations exceeding the regulation standards during April 2018.

Combination	M01	M02	R01	R02	R03	R04	R05	R06	R07	R08	R09	R10	R11	R12
1	F	F	F	F	F	F	F	F	F	F	F	T	F	T
2	F	F	F	F	F	F	F	T	F	F	T	T	F	T
3	F	F	F	F	F	T	F	T	T	F	F	T	F	T
4	F	F	F	F	F	T	F	T	T	F	T	T	F	T
5	F	T	F	F	F	F	F	F	F	F	F	F	F	T
6	F	T	F	F	F	F	F	F	F	F	F	T	F	T
7	F	T	F	F	F	F	F	F	F	F	T	T	F	T
8	F	T	F	F	F	F	F	F	T	F	F	T	F	T
9	F	T	F	F	F	F	F	T	F	F	F	T	F	T
10	F	T	F	F	F	F	F	T	F	F	T	T	F	T
11	F	T	F	F	F	F	F	T	F	T	F	T	F	T
12	F	T	F	F	F	F	F	T	T	F	F	T	F	T
13	F	T	F	F	F	F	F	T	T	F	T	T	F	T
14	F	T	F	F	F	T	F	F	T	F	F	T	F	T
15	F	T	F	F	F	T	F	F	T	F	T	F	F	T
16	F	T	F	F	F	T	F	T	F	F	F	T	F	T
17	F	T	F	F	F	T	F	T	F	F	T	T	F	T
18	F	T	F	F	F	T	F	T	T	F	F	T	F	T
19	F	T	F	F	F	T	F	T	T	F	T	T	F	T
20	F	T	T	F	F	T	F	T	T	F	F	T	F	T
21	F	T	T	F	F	T	F	T	T	F	T	T	F	T
22	T	F	F	F	F	T	F	T	F	F	T	T	F	T
23	T	F	T	F	F	T	F	T	F	F	T	T	F	T
24	T	T	F	F	F	F	F	F	F	F	F	T	F	T
25	T	T	F	F	F	F	F	T	F	F	T	T	F	T
26	T	T	F	F	F	T	F	F	F	F	F	T	F	T
27	T	T	F	F	F	T	F	T	F	F	F	T	F	T
28	T	T	F	F	F	T	F	T	F	F	T	T	F	T
29	T	T	T	F	F	T	F	T	F	F	F	T	F	T
30	T	T	T	F	F	T	F	T	F	F	T	T	F	T

Note: T refers to monitoring values that exceed regulation standard; F refers to monitoring values that do not exceed regulation standards.

Table S9. Groups with combinations of Cu concentrations exceeding the regulation standards during the study time period.

Combination	M01	M02
1	F	F
2	F	T

Note: T refers to monitoring values that exceed regulation standard; F refers to monitoring values that do not exceed regulation standards.

Table S10. Data upload procedures of Case 1

**EC**
Step 1：Operation at R04<br>Step 1-1: Since there are no coins at the address of this station, the Issuer sends a coin to the station address. (Issuer -> ['R04'])<br>Step 1-2: The upstream area is checked and R06 is detected to be polluted; it is therefore considered that this pollution event originates at R06 and affects R04. The coin is thus sent to R06. (R04 -> ['R06'])<br>Step 2：Operation at R06<br>Step 2-1: Since there are coins at the address of this station, there is no need for the Issuer to send a coin to this station.<br>Step 2-2: The upstream area is checked and M01 is detected to be polluted; it is therefore considered that this pollution event originates at M01 and affects R06. The coin is thus sent to M01. (R06 -> ['M01'])<br>Step 3：Operation at M01<br>Step 3-1: Since there are coins at the address of this station, there is no need for the Issuer to send a coin to this station.<br>Step 3-2: The upstream area is checked and there is no station polluted; it is therefore considered that M01 is the source of this pollution event and the coin is sent to the collection address. (M01 -> [])<br>Step 4：Operation at R09<br>Step 4-1: Since there are no coins at the address of this station, the Issuer sends a coin to the station address. (Issuer -> ['R09'])<br>Step 4-2: The upstream area is checked and there is no station polluted; it is therefore considered that R09 is the source of this pollution event and a coin is sent to the collection address. (R09 -> [])<br>Step 5：Operation at R10<br>Step 5-1: Since there are no coins at the address of this station, the Issuer sends a coin to the station address. (Issuer -> ['R10'])<br>Step 5-2: The upstream area is checked and there is no station polluted; it is therefore considered that R10 is the source of this pollution event and the coin is sent to the collection address. (R10 -> [])<br>Step 6：Operation at R12<br>Step 6-1: Since there are no coins at the address of this station, the Issuer sends a coin to the station address. (Issuer -> ['R12'])<br>Step 6-2: The upstream area is checked and there is no station polluted; it is therefore considered that R12 is the source of this pollution event and the coin is sent to the collection address. (R12 -> [])
**Cu**
Step 1：Operation at M02

Step 1-1: Since there are no coins at the address of this station, the Issuer sends a coin to the station address. (Issuer -> ['M02'])

Step 1-2: The upstream area is checked and there is no station polluted; it is therefore considered that M02 is the source of this pollution event and the coin is sent to the collection address. (M02 -> [])

2018-04-26 15:00:00

Table S11. Data upload procedures of Case 2

**EC**
Step 1：Operation at R04<br>Step 1-1: Since there are no coins at the address of this station, the Issuer sends a coin to the station address. (Issuer -> ['R04'])<br>Step 1-2: The upstream area is checked and R06 is detected to be polluted; it is therefore considered that this pollution event originates at R06 and affects R04. The coin is thus sent to R06. (R04 -> ['R06'])<br>Step 2：Operation at R06<br>Step 2-1: Since there are coins at the address of this station, there is no need for the Issuer to send a coin to this station.<br>Step 2-2: The upstream area is checked and M01 is detected to be polluted; it is therefore considered that this pollution event originates at M01 and affects R06. The coin is thus sent to M01. (R06 -> ['M01'])<br>Step 3：Operation at M01<br>Step 3-1: Since there are coins at the address of this station, there is no need for the Issuer to send a coin to this station.<br>Step 3-2: The upstream area is checked and there is no station polluted; it is therefore considered that M01 is the source of this pollution event and the coin is sent to the collection address. (M01 -> [])<br>Step 4：Operation at R09<br>Step 4-1: Since there are no coins at the address of this station, the Issuer sends a coin to the station address. (Issuer -> ['R09'])<br>Step 4-2: The upstream area is checked and there is no station polluted; it is therefore considered that R09 is the source of this pollution event and the coin is sent to the collection address. (R09 -> [])<br>Step 5：Operation at R10<br>Step 5-1: Since there are no coins at the address of this station, the Issuer sends a coin to the station address. (Issuer -> ['R10'])<br>Step 5-2: The upstream area is checked and there is no station polluted; it is therefore considered that R10 is the source of this pollution event and the coin is sent to the collection address. (R10 -> [])<br>Step 6：Operation at R12<br>Step 6-1: Since there are no coins at the address of this station, the Issuer sends a coin to the station address. (Issuer -> ['R12'])<br>Step 6-2: The upstream area is checked and there is no station polluted; it is therefore considered that R12 is the source of this pollution event and the coin is sent to the collection address. (R12 -> [])
**Cu**
Step 1：Operation at M02<br>Step 1-1: Since there are no coins at the address of this station, the Issuer sends a coin to the station address. (Issuer -> ['M02'])<br>Step 1-2: The upstream area is checked and there is no station polluted; it is therefore considered that M02 is the source of this pollution event and the coin is sent to the collection address. (M02 -> [])<br>2018-04-26 20:00:00

Table S12. Data upload procedures of Case 3

**EC**
Step 1：Operation at R04<br>Step 1-1: Since there are no coins at the address of this station, the Issuer sends a coin to the station address. (Issuer -> ['R04'])

Step 1-2: The upstream area is checked and R06 is detected to be polluted; it is therefore considered that this pollution event originates at R06 and affects R04. The coin is thus sent to R06. (R04 -> ['R06'])

Step 2：Operation at R06

Step 2-1: Since there are coins at the address of this station, there is no need for the Issuer to send a coin to this station.

Step 2-2: The upstream area is checked and M01 is detected to be polluted; it is therefore considered that this pollution event originates M01 and affects R06. The coin is thus sent to M01. (R06 -> ['M01'])

Step 3：Operation at M01

Step 3-1: Since there are coins at the address of this station, there is no need for the Issuer to send a coin to this station.

Step 3-2: The upstream area is checked and there is no station polluted; it is therefore considered that M01 is the source of this pollution event and the coin is sent to the collection address. (M01 -> [])

Step 4：Operation at R09

Step 4-1: Since there are no coins at the address of this station, the Issuer sends a coin to the station address. (Issuer -> ['R09'])

Step 4-2: The upstream area is checked and there is no station polluted; it is therefore considered that R09 is the source of this pollution event and the coin is sent to the collection address. (R09 -> [])

Step 5：Operation at R10

Step 5-1: Since there are no coins at the address of this station, the Issuer sends a coin to the station address. (Issuer -> ['R10'])

Step 5-2: The upstream area is checked and there is no station polluted; it is therefore considered that R10 is the source of this pollution event and the coin is sent to the collection address. (R10 -> [])

Step 6：Operation at R12

Step 6-1: Since there are no coins at the address of this station, the Issuer sends a coin to the station address. (Issuer -> ['R12'])

Step 6-2: The upstream area is checked and there is no station polluted; it is therefore considered that R12 is the source of this pollution event and the coin is sent to the collection address. (R12 -> [])

Cu

Step 1：Operation at M02

Step 1-1: Since there are no coins at the address of this station, the Issuer sends a coin to the station address. (Issuer -> ['M02'])

Step 1-2: The upstream area is checked and there is no station polluted; it is therefore considered that M02 is the source of this pollution event and the coin is sent to the collection address. (M02 -> [])

2018-04-27 01:00:00

Table S13. Data upload procedures of Case 4

EC

Step 1：Operation at R04

Step 1-1: Since there are no coins at the address of this station, the Issuer sends a coin to the station address. (Issuer -> ['R04'])

Step 1-2: The upstream area is checked and R06 is detected to be polluted; it is therefore considered that this pollution event originates at R06 and affects R04. The coin is thus sent to R06. (R04 -> ['R06'])

Step 2：Operation at R06

Step 2-1: Since there are coins at the address of this station, there is no need for the Issuer to send a coin to this station.

Step 2-2: The upstream area is checked and M01 is detected to be polluted; it is therefore considered that this pollution event originates at M01 and affects R06. The coin is thus sent to M01. (R06 -> ['M01'])<br>Step 3：Operation at M01<br>Step 3-1: Since there are coins at the address of this station, there is no need for the Issuer to send a coin to this station.<br>Step 3-2: The upstream area is checked and there is no station polluted; it is therefore considered that M01 is the source of this pollution event and the coin is sent to the collection address. (M01 -> [])<br>Step 4：Operation at R09<br>Step 4-1: Since there are no coins at the address of this station, the Issuer sends a coin to the station address. (Issuer -> ['R09'])<br>Step 4-2: The upstream area is checked and there is no station polluted; it is therefore considered that R09 is the source of this pollution event and the coin is sent to the collection address. (R09 -> [])<br>Step 5：Operation at R10<br>Step 5-1: Since there are no coins at the address of this station, the Issuer sends a coin to the station address. (Issuer -> ['R10'])<br>Step 5-2: The upstream area is checked and M02 is detected to be polluted; it is therefore considered that this pollution event originates at M02 and affects R10. The coin is thus sent to M02. (R10 -> ['M02'])<br>Step 6：Operation at R12<br>Step 6-1: Since there are no coins at the address of this station, the Issuer sends a coin to the station address. (Issuer -> ['R12'])<br>Step 6-2: The upstream area is checked and M02 is detected to be polluted; it is therefore considered that this pollution event originates at M02 and affects R12. The coin is thus sent to M02. (R12 -> ['M02'])<br>Step 7：Operation at M02<br>Step 7-1: Since there are coins at the address of this station, there is no need for the Issuer to send a coin to this station.<br>Step 7-2: The upstream area is checked and there is no station polluted; it is therefore considered that M02 is the source of this pollution event and the coin is sent to the collection address. (M02 -> [])<br>2018-04-27 06:00:00

Table S14. Data upload procedures of Case 5

**EC**
Step 1：Operation at R04<br>Step 1-1: Since there are no coins at the address of this station, the Issuer sends a coin to the station address. (Issuer -> ['R04'])<br>Step 1-2: The upstream area is checked and R06 is detected to be polluted; it is therefore considered that this pollution event originates at R06 and affects R04. The coin is thus sent to R06. (R04 -> ['R06'])<br>Step 2：Operation at R06<br>Step 2-1: Since there are coins at the address of this station, there is no need for the Issuer to send a coin to this station.<br>Step 2-2: The upstream area is checked and M01 is detected to be polluted; it is therefore considered that this pollution event originates at M01 and affects R06. The coin is thus sent to M01. (R06 -> ['M01'])<br>Step 3：Operation at M01<br>Step 3-1: Since there are coins at the address of this station, there is no need for the Issuer to send a coin to this station.

Step 3-2: The upstream area is checked and there is no station polluted; it is therefore considered that M01 is the source of this pollution event and the coin is sent to the collection address. (M01 -> [])<br>Step 4：Operation at R09<br>Step 4-1: Since there are no coins at the address of this station, the Issuer sends a coin to the station address. (Issuer -> ['R09'])<br>Step 4-2: The upstream area is checked and there is no station polluted; it is therefore considered that R09 is the source of this pollution event and the coin is snet to the collection address. (R09 -> [])<br>Step 5：Operation at R10<br>Step 5-1: Since there are no coins at the address of this station, the Issuer sends a coin to the station address. (Issuer -> ['R10'])<br>Step 5-2: The upstream area is checked and M02 is detected to be polluted; it is therefore considered that this pollution event originates at M02 and affects R10. The coin is thus sent to M02. (R10 -> ['M02'])<br>Step 6：Operation at R12<br>Step 6-1: Since there are no coins at the address of this station, the Issuer sends a coin to the station address. (Issuer -> ['R12'])<br>Step 6-2: The upstream area is checked and M02 is detected to be polluted; it is therefore considered that this pollution event originates at M02 and affects R12. The coin is thus sent to M02. (R12 -> ['M02'])<br>Step 7：Operation at M02<br>Step 7-1: Since there are coins at the address of this station, there is no need for the Issuer to send a coin to this station.<br>Step 7-2: The upstream area is checked and there is no station polluted; it is therefore considered that M02 is the source of this pollution event and the coin is sent to the collection address. (M02 -> [])<br>2018-04-27 17:00:00

Table S15. Data upload procedures of Case 6

**EC**
Step 1：Operation at R04<br>Step 1-1: Since there are no coins at the address of this station, the Issuer sends a coin to the station address. (Issuer -> ['R04'])<br>Step 1-2: The upstream area is checked and R06 is detected to be polluted; it is therefore considered that this pollution event originates at R06 and affects R04. The coin is thus sent to R06. (R04 -> ['R06'])<br>Step 2：Operation at R06<br>Step 2-1: Since there are coins at the address of this station, there is no need for the Issuer to send a coin to this station.<br>Step 2-2: The upstream area is checked and M01 is detected to be polluted; it is therefore considered that this pollution event originates at M01 and affects R06. The coin is thus sent to M01. (R06 -> ['M01'])<br>Step 3：Operation at M01<br>Step 3-1: Since there are coins at the address of this station, there is no need for the Issuer to send a coin to this station.<br>Step 3-2: The upstream area is checked and there is no station polluted; it is therefore considered that M01 is the source of this pollution event and the coin is sent to the collection address. (M01 -> [])<br>Step 4：Operation at R09<br>Step 4-1: Since there are no coins at the address of this station, the Issuer sends a coin to the station address. (Issuer -> ['R09'])

Step 4-2: The upstream area is checked and there is no station polluted; it is therefore considered that R09 is the source of this pollution event and the coin is sent to the collection address. (R09 -> [])   Step 5：Operation at R10   Step 5-1: Since there are no coins at the address of this station, the Issuer sends a coin to the station address. (Issuer -> ['R10'])   Step 5-2: The upstream area is checked and M02 is detected to be polluted; it is therefore considered that this pollution event originates at M02 and affects R10. The coin is thus sent to M02. (R10 -> ['M02'])   Step 6：Operation at R12   Step 6-1: Since there are no coins at the address of this station, the Issuer sends a coin to the station address. (Issuer -> ['R12'])   Step 6-2: The upstream area is checked and M02 is detected to be polluted; it is therefore considered that this pollution event originates at M02 and affects R12. The coin is thus sent to M02. (R12 -> ['M02'])   Step 7：Operation at M02   Step 7-1: Since there are coins at the address of this station, there is no need for the Issuer to send a coin to this station.   Step 7-2: The upstream area is checked and there is no station polluted; it is therefore considered that M02 is the source of this pollution event and the coin is sent to the collection address. (M02 -> [])
**Cu**
Step 1：Operation at M02   Step 1-1: Since there are no coins at the address of this station, the Issuer sends a coin to the station address. (Issuer -> ['M02'])   Step 1-2: The upstream area is checked and there is no station polluted; it is therefore considered that M02 is the source of this pollution event and the coin is sent to the collection address. (M02 -> [])   2018-04-27 22:00:00

Table S16. Data upload procedures of Case 7

**EC**
Step 1：Operation at R04   Step 1-1: Since there are no coins at the address of this station, the Issuer sends a coin to the station address. (Issuer -> ['R04'])   Step 1-2: The upstream area is checked and R06 is detected to be polluted; it is therefore considered that this pollution event originates at R06 and affects R04. The coin is thus sent to R06. (R04 -> ['R06'])   Step 2：Operation at R06   Step 2-1: Since there are coins at the address of this station, there is no need for the Issuer to send a coin to this station.   Step 2-2: The upstream area is checked and M01 is detected to be polluted; it is therefore considered that this pollution event originates at M01 and affects R06. The coin is thus sent to M01. (R06 -> ['M01'])   Step 3：Operation at M01   Step 3-1: Since there are coins at the address of this station, there is no need for the Issuer to send a coin to this station.   Step 3-2: The upstream area is checked and there is no station polluted; it is therefore considered that M01 is the source of this pollution event and the coin is sent to the collection address. (M01 -> [])   Step 4：Operation at R09   Step 4-1: Since there are no coins at the address of this station, the Issuer sends a coin to the station address. (Issuer -> ['R09'])

Step 4-2: The upstream area is checked and there is no station polluted; it is therefore considered that R09 is the source of this pollution event and the coin is sent to the collection address. (R09 -> [])<br>Step 5：Operation at R10<br>Step 5-1: Since there are no coins at the address of this station, the Issuer sends a coin to the station address. (Issuer -> ['R10'])<br>Step 5-2: The upstream area is checked and M02 is detected to be polluted; it is therefore considered that this pollution event originates at M02 and affects R10. The coin is thus sent to M02. (R10 -> ['M02'])<br>Step 6：Operation at R12<br>Step 6-1: Since there are no coins at the address of this station, the Issuer sends a coin to the station address. (Issuer -> ['R12'])<br>Step 6-2: The upstream area is checked and M02 is detected to be polluted; it is therefore considered that this pollution event originates at M02 and affects R12. The coin is thus sent to M02. (R12 -> ['M02'])<br>Step 7：Operation at M02<br>Step 7-1: Since there are coins at the address of this station, there is no need for the Issuer to send a coin to this station.<br>Step 7-2: The upstream area is checked and there is no station polluted; it is therefore considered that M02 is the source of this pollution event and the coin is sent to the collection address. (M02 -> [])
**Cu**
Step 1：Operation at M02<br>Step 1-1: Since there are no coins at the address of this station, the Issuer sends a coin to the station address. (Issuer -> ['M02'])<br>Step 1-2: The upstream area is checked and there is no station polluted; it is therefore considered that M02 is the source of this pollution event and the coin is sent to the collection address. (M02 -> [])<br>2018-04-28 23:00:00

Table S17. Data upload procedures of Case 8

**EC**
Step 1：Operation at R04<br>Step 1-1: Since there are no coins at the address of this station, the Issuer sends a coin to the station address. (Issuer -> ['R04'])<br>Step 1-2: The upstream area is checked and R06 is detected to be polluted; it is therefore considered that this pollution event originates at R06 and affects R04. The coin is thus sent to R06. (R04 -> ['R06'])<br>Step 2：Operation at R06<br>Step 2-1: Since there are coins at the address of this station, there is no need for the Issuer to send a coin to this station.<br>Step 2-2: The upstream area is checked and M01 is detected to be polluted; it is therefore considered that this pollution event originates at M01 and affects R06. The coin is thus sent to M01. (R06 -> ['M01'])<br>Step 3：Operation at M01<br>Step 3-1: Since there are coins at the address of this station, there is no need for the Issuer to send a coin to this station.<br>Step 3-2: The upstream area is checked and there is no station polluted; it is therefore considered that M01 is the source of this pollution event and the coin is sent to the collection address. (M01 -> [])<br>Step 4：Operation at R09<br>Step 4-1: Since there are no coins at the address of this station, the Issuer sends a coin to the station address. (Issuer -> ['R09'])

Step 4-2: The upstream area is checked and there is no station polluted; it is therefore considered that R09 is the source of this pollution event and the coin is sent to the collection address. (R09 -> [])<br>Step 5：Operation at R10<br>Step 5-1: Since there are no coins at the address of this station, the Issuer sends a coin to the station address. (Issuer -> ['R10'])<br>Step 5-2: The upstream area is checked and M02 is detected to be polluted; it is therefore considered that this pollution event originates at M02 and affects R10. The coin is thus sent to M02. (R10 -> ['M02'])<br>Step 6：Operation at R12<br>Step 6-1: Since there are no coins at the address of this station, the Issuer sends a coin to the station address. (Issuer -> ['R12'])<br>Step 6-2: The upstream area is checked and M02 is detected to be polluted; it is therefore considered that this pollution event originates at M02 and affects R12. The coin is thus sent to M02. (R12 -> ['M02'])<br>Step 7：Operation at M02<br>Step 7-1: Since there are coins at the address of this station, there is no need for the Issuer to send a coin to this station.<br>Step 7-2: The upstream area is checked and there is no station polluted; it is therefore considered that M02 is the source of this pollution event and the coin is sent to the collection address. (M02 -> [])
**Cu**
Step 1：Operation at M02<br>Step 1-1: Since there are no coins at the address of this station, the Issuer sends a coin to the station address. (Issuer -> ['M02'])<br>Step 1-2: The upstream area is checked and there is no station polluted; it is therefore considered that M02 is the source of this pollution event and the coin is sent to the collection address. (M02 -> [])<br>2018-04-29 13:00:00

Table S18. Data upload procedures of Case 9

**EC**
Step 1：Operation at R04<br>Step 1-1: Since there are no coins at the address of this station, the Issuer sends a coin to the station address. (Issuer -> ['R04'])<br>Step 1-2: The upstream area is checked and R06 is detected to be polluted; it is therefore considered that this pollution event originats at R06 and affects R04. The coin is thus sent to R06. (R04 -> ['R06'])<br>Step 2：Operation at R06<br>Step 2-1: Since there are coins at the address of this station, there is no need for the Issuer to send a coin to this station.<br>Step 2-2: The upstream area is checked and M01 is detected to be polluted; it is therefore considered that this pollution event originates at M01 and affects R06. The coin is thus sent to M01. (R06 -> ['M01'])<br>Step 3：Operation at M01<br>Step 3-1: Since there are coins at the address of this station, there is no need for the Issuer to send a coin to this station.<br>Step 3-2: The upstream area is checked and there is no station polluted; it is therefore considered that M01 is the source of this pollution event and the coin is sent to the collection address. (M01 -> [])<br>Step 4：Operation at R09<br>Step 4-1: Since there are no coins at the address of this station, the Issuer sends a coin to the station address. (Issuer -> ['R09'])

Step 4-2: The upstream area is checked and there is no station polluted; it is therefore considered that R09 is the source of this pollution event and the coin is sent to the collection address. (R09 -> [])<br>Step 5：Operation at R10<br>Step 5-1: Since there are no coins at the address of this station, the Issuer sends a coin to the station address. (Issuer -> ['R10'])<br>Step 5-2: The upstream area is checked and M02 is detected to be polluted; it is therefore considered that this pollution event originates at M02 and affects R10. The coin is thus sent to M02. (R10 -> ['M02'])<br>Step 6：Operation at R12<br>Step 6-1: Since there are no coins at the address of this station, the Issuer sends a coin to the station address. (Issuer -> ['R12'])<br>Step 6-2: The upstream area is checked and M02 is detected to be polluted; it is therefore considered that this pollution event originates at M02 and affects R12. The coin is thus sent to M02. (R12 -> ['M02'])<br>Step 7：Operation at M02<br>Step 7-1: Since there are coins at the address of this station, there is no need for the Issuer to send a coin to this station.<br>Step 7-2: The upstream area is checked and there is no station polluted; it is therefore considered that M02 is the source of this pollution event and the coin is sent to the collection address. (M02 -> [])
**Cu**
Step 1：Operation at M02<br>Step 1-1: Since there are no coins at the address of this station, the Issuer sends a coin to the station address. (Issuer -> ['M02'])<br>Step 1-2: The upstream area is checked and there is no station polluted; it is therefore considered that M02 is the source of this pollution event and the coin is sent to the collection address. (M02 -> [])<br>2018-04-29 19:00:00

Table S19. Data upload procedures of Case 10

**EC**
Step 1：Operation at R04<br>Step 1-1: Since there are no coins at the address of this station, the Issuer sends a coin to the station address. (Issuer -> ['R04'])<br>Step 1-2: The upstream area is checked and R06 is detected to be polluted; it is therefore considered that this pollution event originates R06 and affects R04. The coin is thus sent to R06. (R04 -> ['R06'])<br>Step 2：Operation at R06<br>Step 2-1: Since there are coins at the address of this station, there is no need for the Issuer to send a coin to this station.<br>Step 2-2: The upstream area is checked and M01 is detected to be polluted; it is therefore considered that this pollution event is generated by M01 and affects R06. The coin is thus sent to M01. (R06 -> ['M01'])<br>Step 3：Operation at M01<br>Step 3-1: Since there are coins at the address of this station, there is no need for the Issuer to send a coin to this station.<br>Step 3-2: The upstream area is checked and there is no station polluted; it is therefore considered that M01 is the source of this pollution event and the coin is sent to the collection address. (M01 -> [])<br>Step 4：Operation at R09<br>Step 4-1: Since there are no coins at the address of this station, the Issuer sends a coin to the station address. (Issuer -> ['R09'])

Step 4-2: The upstream area is checked and there is no station polluted; it is therefore considered that R09 is the source of this pollution event and the coin is sent to the collection address. (R09 -> [])<br>Step 5：Operation at R10<br>Step 5-1: Since there are no coins at the address of this station, the Issuer sends a coin to the station address. (Issuer -> ['R10'])<br>Step 5-2: The upstream area is checked and M02 is detected to be polluted; it is therefore considered that this pollution event originates at M02 and affects R10. The coin is thus sent to M02. (R10 -> ['M02'])<br>Step 6：Operation at R12<br>Step 6-1: Since there are no coins at the address of this station, the Issuer sends a coin to the station address. (Issuer -> ['R12'])<br>Step 6-2: The upstream area is checked and M02 is detected to be polluted; it is therefore considered that this pollution event originates at M02 and affects R12. The coin is thus sent to M02. (R12 -> ['M02'])<br>Step 7：Operation at M02<br>Step 7-1: Since there are coins at the address of this station, there is no need for the Issuer to send a coin to this station.<br>Step 7-2: The upstream area is checked and there is no station polluted; it is therefore considered that M02 is the source of this pollution event and the coin is sent to the collection address. (M02 -> [])
**Cu**
Step 1：Operation at M02<br>Step 1-1: Since there are no coins at the address of this station, the Issuer sends a coin to the station address. (Issuer -> ['M02'])<br>Step 1-2: The upstream area is checked and there is no station polluted; it is therefore considered that M02 is the source of this pollution event and the coin is sent to the collection address. (M02 -> [])<br>2018-04-30 00:00:00

References

1. Taiwan Council of Agriculture, 2016. The Establishment of Classified Management of Water and Soil Resources and Early Warning Mechanism, Technical Report.

2. Wool, T. A., Ambrose, R. B., Martin, J. L., & Comer, E. A. (2003). Draft: User's manual. Water quality simulation WASP. Atlanta: USEPA. (https://www.epa.gov/ceam/modeling-products-assess-exposures).

3. Delurgio, Forecasting Principle and Application, McGraw-Hill, New Jersey, 1988.

4. Huang K.-T., 2019, Apply Blockchain Technology To Irrigation Water Quality Recording And Pollution Traceability: A Case Study On Taoyuan Canal Distributary 2, 3, 4, Master Thesis.
